# Total synthesis of pyrrolo[2,3-*c*]quinoline alkaloid: trigonoine B

**DOI:** 10.3762/bjoc.17.62

**Published:** 2021-03-16

**Authors:** Takashi Nishiyama, Erina Hamada, Daishi Ishii, Yuuto Kihara, Nanase Choshi, Natsumi Nakanishi, Mari Murakami, Kimiko Taninaka, Noriyuki Hatae, Tominari Choshi

**Affiliations:** 1 Faculty of Pharmacy and Pharmaceutical Sciences, Fukuyama University, Fukuyama, Hiroshima 729-0292, Japan; 2 Faculty of Pharmaceutical Sciences, Yokohama University of Pharmacy, 601 Matano, Totsuka-ku, Yokohama 245-0066, Japan

**Keywords:** 2-azahexatiriene system, carbodiimide, electrocyclization, pyrrolo[2,3-*c*]quinoline, trigonoine B

## Abstract

The first total synthesis of the pyrrolo[2,3-*c*]quinoline alkaloid trigonoine B (**1**) was accomplished via a six-step sequence involving the construction of an *N*-substituted 4-aminopyrrolo[2,3-*c*]quinoline framework via electrocyclization of 2-(pyrrol-3-yl)benzene containing a carbodiimide moiety as a 2-azahexatriene system. The employed six-step sequence afforded trigonoine B (**1**) in 9.2% overall yield. The described route could be employed for the preparation of various *N*-substituted 4-aminopyrroloquinolines with various biological activities.

## Introduction

**Figure 1:**
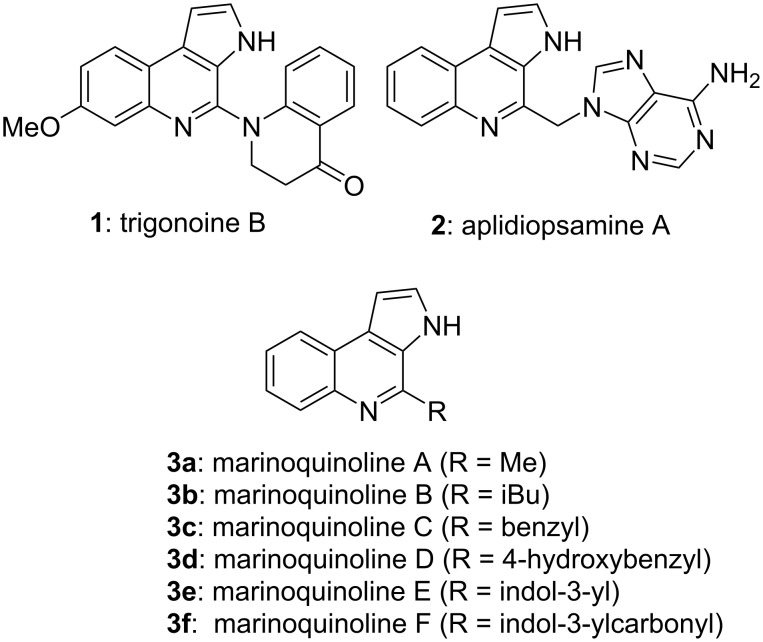
Natural products possessing the pyrrolo[2,3-*c*]quinoline skeleton.

In 2011, two novel alkaloids, namely trigonoine A and B, were isolated from the leaves of *Trigonostemon lii* by Hao and co-workers [1]. The structures of the compounds were elucidated through 1D and 2D NMR spectroscopy. It was determined that trigonoine A was a β-carboline alkaloid containing a 2,8-diazabicyclo[3.3.1]nonane ring system, while trigonoine B (**1**) was formed by a combination of 2,3-dihydroquinolin-4-one and 3*H*-pyrrolo[2,3-*c*]quinoline rearranged from a β-carboline skeleton (Figure 1).

Additionally, the antihepatitis B virus (HBV) activity of the alkaloids was evaluated in vitro using an HBV-transfected HepG2 cell line. The antihuman immunodeficiency virus (HIV) properties were also investigated to determine whether trigonoine A and B could prevent the cytopathic effects of HIV-1_IIIB_ in C8166 cells. Notably, it was found that both natural products exhibited anti-HIV activity.

In addition to trigonoine B, aplidiopsamine A (**2**) [2] and marinoquinolines **3** [3] are natural products that possess the pyrrolo[2,3-*c*]quinoline skeleton (Figure 1). These pyrrolo[2,3-*c*]quinolines have been demonstrated to show antimalarial and antibacterial biological activities [2–4]. Thus, the development of a convenient and efficient synthetic route to the pyrrolo[2,3-*c*]quinoline skeleton has attracted considerable attention from organic and medicinal chemists. The total syntheses of aplidiopsamine A (**2**) [5–9] and marinoquinolines **3** [10–13] have been achieved by various synthetic strategies. Nevertheless, the total synthesis of trigonoine B (**1**) has not yet been reported.

We have been interested in the synthesis of heterocyclic compounds by constructing fused pyridine ring systems based on a thermal electrocyclization of an azahexatriene moiety [14–15]. It has been hoped that the development of compounds with enhanced biological activity would be possible using these natural products and their derivatives [16–18]. We have previously reported the total syntheses of indolo[3,2-*c*]quinoline (isocryptolepine) [19], azaanthracenones (kalasinamide, marcanine A, and geovanine) [20], imidazo[4',5':4,5]pyrido[2,3-*b*]indole (grossularine-1 and -2) [21–22], imidazo[4,5-*b*]pyridine (2-amino-1-methyl-6-phenylimidazo[4,5-*b*]pyridine and 2-amino-1,6-dimethylimidazo[4,5-*b*]pyridine) [23], and imidazo[4,5-*c*]quinoline (imiquimod) [24] based on the electrocyclization of 2-azahexatriene involving an isocyanate moiety as the key intermediate.

**Scheme 1:**
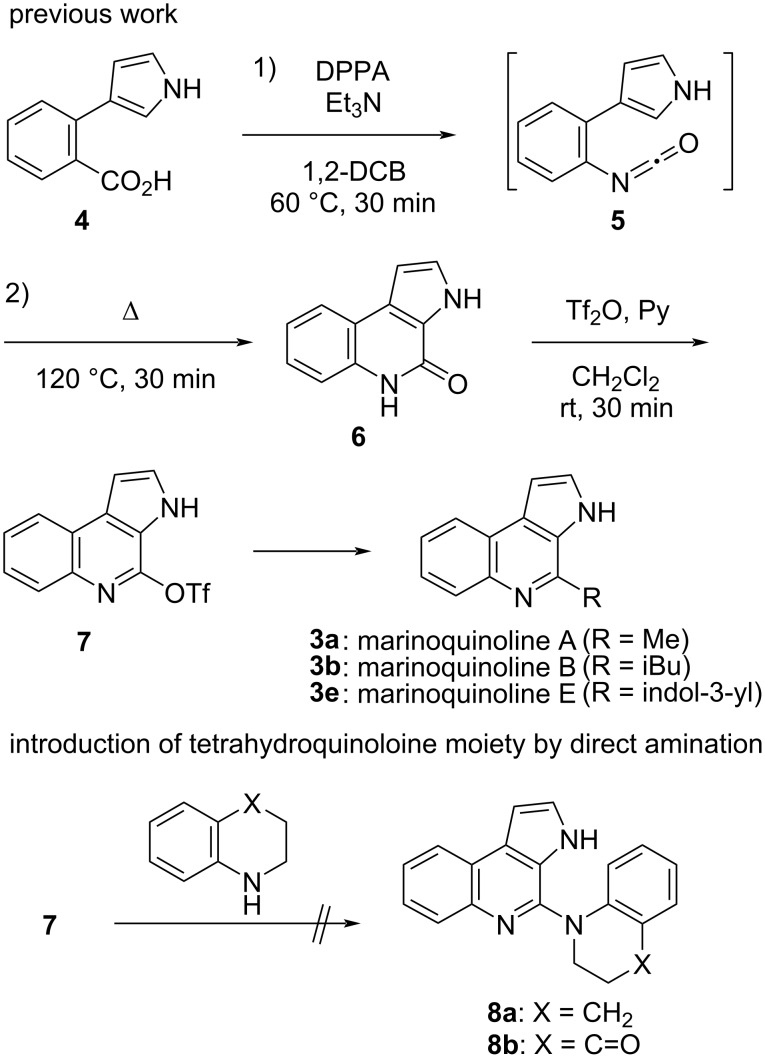
Total synthesis of marinoquinolines and the failure of the introduction of a tetrahydroquinoline moiety by direct amination to triflate **7**.

In addition, we recently reported the total syntheses of marinoquinolines A (**3a**), B (**3b**), and E (**3e**) comprising the pyrrolo[2,3-*c*]quinoline skeleton [25]. As demonstrated in Scheme 1, Curtius rearrangement of carboxylic acid **4** resulted in the formation of isocyanate **5**, which, upon electrocyclization furnished pyrrolo[2,3-*c*]quinoline **6**. Intermediate **6** was subsequently transformed into triflate **7**, and the total syntheses of marinoquinolines **3a**, **3b**, and **3e** were accomplished by introducing different substituents at the C4 position. However, despite our efforts to introduce dihydroquinoline derivatives into triflate **7** using various conditions, the synthesis of **8** could not be achieved. Consequently, we decided to develop a new synthetic strategy for the preparation of trigonoine B (**1**).

Carbodiimides are valuable synthetic intermediates that can be obtained by an aza-Wittig reaction of isocyanates with iminophosphoranes or by dehydration of urea [26]. Molina et al. previously reported the synthesis of 2-aminopyridine derivatives through an electrocyclization of conjugated carbodiimides derived from an aza-Wittig reaction of iminophosphoranes and isocyanates [27]. Hibino and co-workers also achieved the preparation of mutagenic amino-α-carbolines (2-amino-9*H*-pyrido[2,3-*b*]indole, AαC) and MeAαC by the electrocyclization of 3-alkenyl-2-carbodiimidoindole derivatives obtained by an aza-Wittig reaction of indol-2-yl-iminophosphoranes and isocyanates [28].

In this work, we report the first total synthesis of trigonoine B (**1**) involving the construction of a pyrrolo[2,3-*c*]quinoline framework by electrocyclization of 2-(pyrrol-3-yl)benzene containing a carbodiimide moiety as a 2-azahexatriene system.

## Results and Discussion

**Scheme 2:**
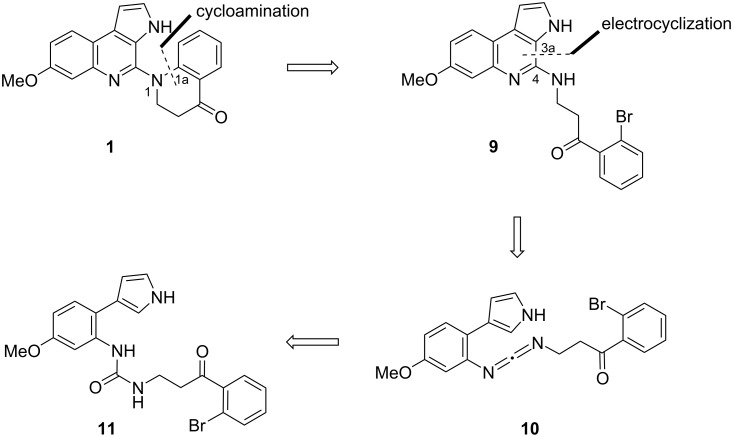
Retrosynthetic analysis of the pyrrolo[2,3-*c*]quinoline ring construction.

Scheme 2 illustrates the retrosynthetic strategy designed to synthesize triogonoine B (**1**). It was speculated that the dihydroquinoline moiety of trigonoine B (**1**) could be constructed through a cycloamination reaction between positions C1a and N1. The synthesis of the precursor pyrroloquinoline **9** possessing a substituted amino group at the 4-position could be achieved by the electrocyclization of pyrrol-3-ylbenzene **10** containing a carbodiimide moiety as a 2-azahexatriene system. Lastly, it was proposed that the carbodiimide **10** could be derived from urea **11**.

**Scheme 3:**
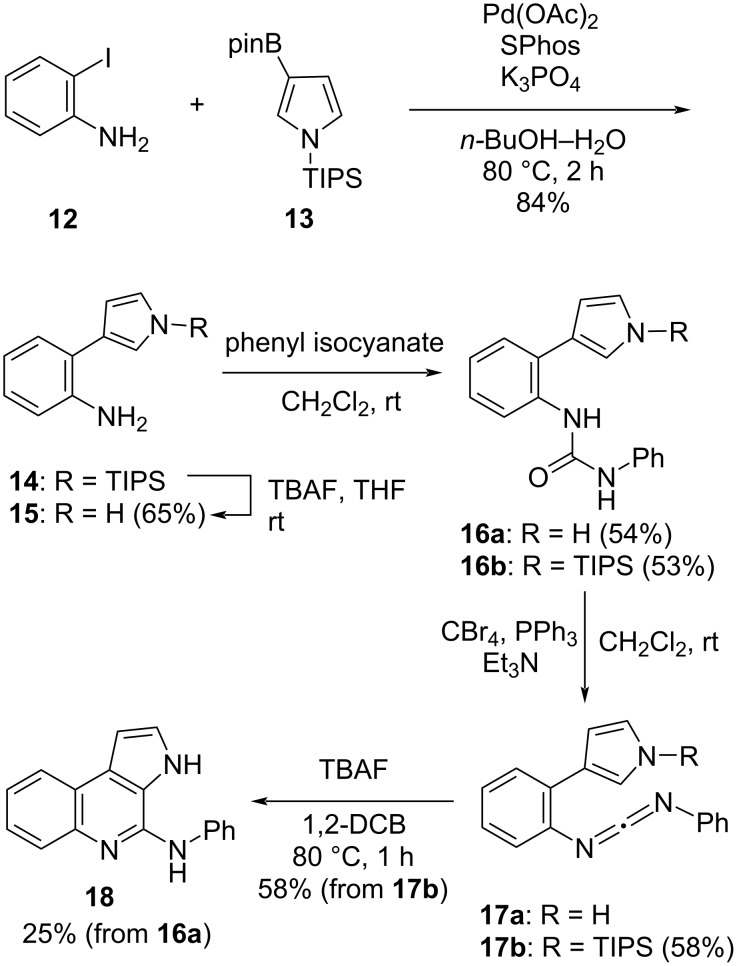
Synthesis of *N*-substituted 4-aminopyrrolo[3,2-*c*]quinoline **18**.

Therefore, we investigated the electrocyclization of a pyrrol-3-ylbenzene containing a carbodiimide moiety. First, 2-(pyrrol-3-yl)aniline **14** was synthesized by a Suzuki–Miyaura coupling reaction of 2-iodoaniline (**12**) and 3-(4,4,5,5-tetramethyl-1,3,2-dioxaborolan-2-yl)-1-[tris(1-methylethyl)silyl]-1*H*-pyrrole (**13**) according to Pratt’s conditions (Scheme 3) [29]. Subsequently, to remove the triisopropylsilyl (TIPS) protecting group, compound **14** was treated with tetra-*n*-butylammonium fluoride (TBAF) in THF, affording aniline **15** in 65% yield. Treatment of **15** with phenyl isocyanate in CH_2_Cl_2_ gave urea **16a** in 54% yield. To obtain carbodiimide **17a**, **16a** was treated with carbon tetrabromide (CBr_4_), PPh_3_, and Et_3_N in CH_2_Cl_2_. The reaction was monitored by TLC, which confirmed the complete consumption of the starting material. However, after the workup, extraction, and removal of solvent (in vacuo), the appearance of the TLC plate changed due to the formation of various byproducts. Then, the reaction mixture was purified, but the desired carbodiimide **17a** was not obtained, but aminopyrroloquinoline **18** formed through electrocyclization of **17a** in 25% yield. Notably, it was found that the electrocyclization of **17a** proceeded easily at low temperature (≈60 °C).

Although we tried to optimize the reaction conditions to improve the yield of **18**, the yield could not be improved owing to the generation of numerous byproducts in the reaction of **16a** with CBr_4_, PPh_3_, and Et_3_N. Using the same procedure, carbodiimide **17b** was then synthesized in 58% yield starting from aniline **14** via urea **16b**. Then, the electrocyclization of **17b** was attempted, but the cyclization did not proceed at all. As possible reason we considered a steric hindrance of the TIPS group. Based on the above results, it was speculated that the electrocyclization of **17b** could proceed continuously following the removal of the TIPS group. Indeed, when **17b** was heated with TBAF in 1,2-dichlorobenzene (1,2-DCB) at 80 °C, the expected reaction proceeded and the desired pyrroloquinoline **18** was obtained in 58% yield.

We subsequently examined the versatility of the electrocyclization of carbodiimides **20a**–**d** as 2-azahexatriene systems (Table 1). First, urea derivatives **19a**–**d** were synthesized by reacting 2-(pyrrol-3-yl)aniline **14** with isocyanates, which were commercially available or prepared from an appropriate carboxylic acid through a Curtius rearrangement reaction (50–98% yield). Treatment of urea derivatives **19a**–**d** with CBr_4_, PPh_3_, and Et_3_N afforded carbodiimides **20a**–**d** in 64–75% yield. Compounds **21a**–**d** were obtained in situ following the removal of the TIPS protecting group in **20a**–**d** by TBAF in 1,2-DCB at 80 °C. The electrocyclization then proceeded immediately, affording the desired pyrroloquinolines **22a**–**c** in 49–90% yield (Table 1, entries 1–3). However, the cyclization of **20d** only gave a mixture of unidentified products (Table 1, entry 4).

**Table 1:** Synthesis of *N*-substituted 4-aminopyrrolo[3,2-*c*]quinolines **22** by electrocyclization of carbodiimides **20**.

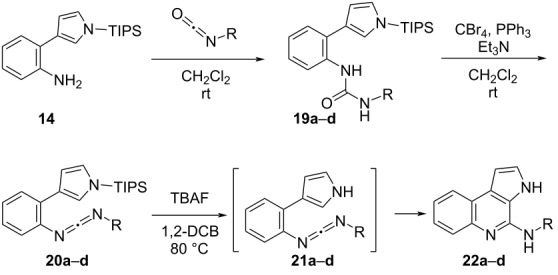								

entry	R		**19**		**20**		**22**	

			time (h)	yield (%)	time (h)	yield (%)	time (h)	yield (%)

1	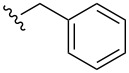	**a**	12	50	4	64	2	90

2	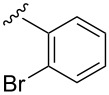	**b**	12	98	1	–^a^	2	49^b^

3	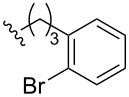	**c**	20	64	2	75	1	61

4	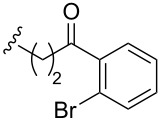	**d**	18	98	0.5	68	1	–^c^

^a^Since carbodiimide **20b** was unstable, the next reaction was carried out without purification. ^b^Yield from **19b**. ^c^Unknown compounds.

**Scheme 4:**
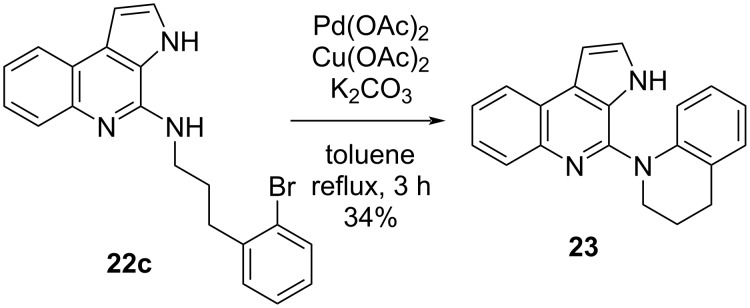
Synthesis of the tetrahydroquinoline moiety through cycloamination.

Hence, we decided to evaluate the synthesis of the 2,3-dihydroquinolin-4-one moiety of trigonoine B (**1**) by cycloamination of **22c** (Scheme 4). The Buchwald–Hartwig amination of **22c** was conducted in the presence of *t-*BuONa, BINAP, and Pd_2_(dba)_3_·CHCl_3_; however, the desired tetrahydroquinoline **23** was not obtained and only **22c** was recovered. We then examined the conditions reported by Orito and co-workers [30]. Gratifyingly, the treatment of **22c** with Pd(OAc)_2_, Cu(OAc)_2_, and K_2_CO_3_ afforded the cyclized product **23** in 34% yield.

**Scheme 5:**
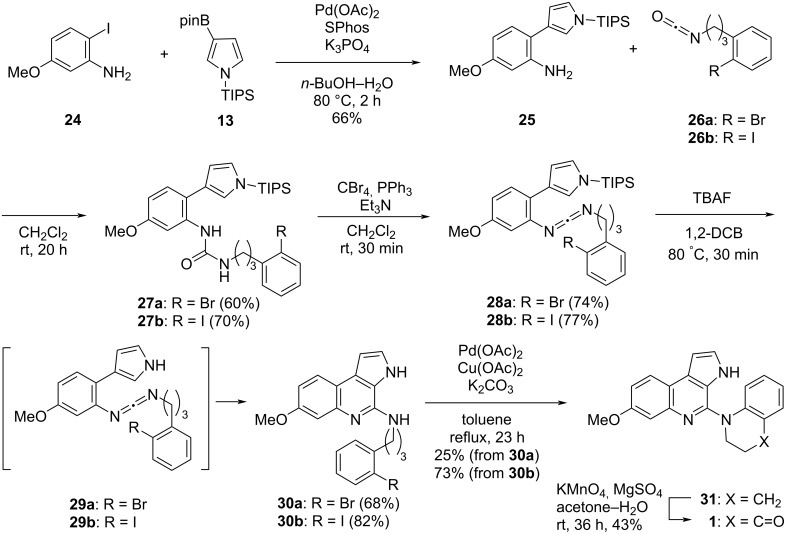
Synthesis of trigonoine B (**1**).

The focus subsequently shifted to the total synthesis of trigonoine B (**1**) (Scheme 5). The key starting material, 2-iodo-5-methoxyaniline (**24**), was synthesized according to the procedure previously reported by Wetzel and co-workers [31]. The Suzuki–Miyaura coupling of 2-iodoaniline derivative **24** and pyrrole-3-boronic acid pinacol ester **13** was carried out in the presence of Pd(OAc)_2_ and SPhos, followed by the treatment of the resulting 2-(pyrrol-3-yl)aniline **25** with 3-(2-bromophenyl)propyl isocyanate (**26a**), which afforded urea **27a** in 60% yield. The treatment of urea **27a** with CBr_4_ and PPh_3_ in the presence of Et_3_N then gave carbodiimide **28a** in a good yield of 74%. Following the reaction of **28a** with TBAF in 1,2-DCB and desilylation, the electrocyclization of **29a** proceeded smoothly to afford the desired 4-aminopyrroloquinoline **30a** in 68% yield. Subsequently, the cycloamination of **30a** in the presence of Pd(OAc)_2_, Cu(OAc)_2_, and K_2_CO_3_ gave tetrahydroquinoline **31** in 25% yield. However, although attempts were made to optimize the cycloamination reaction conditions, the yield could not be improved. We hypothesized that the low yield could be attributed to a too low reactivity of the bromo group. Thus, the same reaction was performed using a compound bearing a more reactive iodo group.

Accordingly, 4-aminopyrroloquinoline **30b** was synthesized from 2-(pyrrol-3-yl)aniline **25** and 3-(2-iodophenyl)propyl isocyanate (**26b**) in 3 steps using the same procedure as described for **30a**. The subsequent cycloamination of **30b** gave tetrahydroquinoline **31** in 73% yield, thus resulting in a significantly improved yield.

Finally, the oxidation of tetrahydroquinoline **31** was examined. Following the evaluation of various reaction conditions [32–34], the best outcome was obtained upon treatment of **31** with KMnO_4_ and MgSO_4_ in an acetone–H_2_O solvent system [35]. Employing these conditions, trigonoine B (**1**) was obtained in 43% yield. The acquired physical and spectroscopic data of the herein synthesized compound **1** were consistent with those of the natural trigonoine B [1]. Thus, in this study, we successfully achieved the first total synthesis of trigonoine B (**1**).

## Conclusion

The total synthesis of a pyrrolo[2,3-*c*]quinoline alkaloid, trigonoine B (**1**), was achieved for the first time through the electrocyclization of 2-(pyrrol-3-yl)benzene bearing a carbodiimide moiety as a 2-azahexatriene system. The employed six-step sequence afforded the desired product **1** in 9.2% overall yield. Notably, the developed synthetic route could be used for the synthesis of various *N*-substituted 4-aminopyrroloquinolines. The investigation of the biological activity of trigonoine B and its derivatives is under evaluation.

## Supporting Information

File 1 ^1^H NMR and ^13^C NMR spectra of all new compounds.
