# Supplementary material for: Total synthesis of pyrrolo[2,3-c]quinoline alkaloid: trigonoine B
Source: Beilstein J Org Chem. 2021 Mar 16;17:730–6. doi: 10.3762/bjoc.17.62 (PMC7991618; doi:10.3762/bjoc.17.62)

BEILSTEIN JOURNAL OF ORGANIC CHEMISTRY

Supporting Information

for

Total synthesis of pyrrolo[2,3-*c*]quinoline alkaloid: trigonoine B

Takashi Nishiyama, Erina Hamada, Daishi Ishii, Yuuto Kihara, Nanase Choshi, Natsumi Nakanishi, Mari Murakami, Kimiko Taninaka, Noriyuki Hatae and Tominari Choshi

Beilstein J. Org. Chem. **2021,** *17,* 730–736. doi:10.3762/bjoc.17.62

^{1}H NMR and ^{13}C NMR spectra of all new compounds

License and Terms: This is a supporting information file under the terms of the Creative Commons Attribution License (https://creativecommons.org/licenses/by/4.0). Please note that the reuse, redistribution and reproduction in particular requires that the author(s) and source are credited and that individual graphics may be subject to special legal provisions.
The license is subject to the *Beilstein Journal of Organic Chemistry* terms and conditions: (https://www.beilstein-journals.org/bjoc/terms)

EXPERIMENTAL SECTION

General experimental details. All non-aqueous reactions were carried out under an atmosphere of nitrogen in dried glassware unless otherwise noted. Solvents were dried and distilled according to standard protocols. Analytical thin-layer chromatography was performed with silica gel $60PF_{254}$ (Merck). Silica gel column chromatography was performed with Silica gel 60 (70–230 mesh, Kanto Chemical Co. Lit.). All melting points were determined on Yanagimoto micro melting point apparatus and are uncorrected. Proton nuclear magnetic resonance (^{1}H NMR) spectra were recorded on a JEOL AL-300 at 300 MHz. Chemical shifts are reported relative to Me_4Si (δ 0.00). Multiplicity is indicated by one or more of the following: s (singlet); d (doublet); t (triplet); q (quartet); quin (quintet); sept (septet); m (multiplet); br (broad). Carbon nuclear magnetic resonance (^{13}C NMR) spectra were recorded on a JEOL AL-300 at 75 MHz. Chemical shifts are reported relative to $CDCl_3$ (δ 77.0) and DMSO-d_6 (δ 39.7). Infrared spectra were recorded with ATR method using a Shimadzu FTIR-8000 spectrophotometer and Technologies DuraScop. Low and high-resolution mass spectra were recorded on JEOL JMS-700 spectrometers by a direct inlet system.

2-(1-Triisopropylsilyl-1*H*-pyrrol-3-yl)aniline (14)[1]

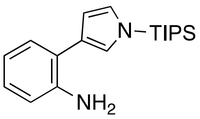

The synthesis of the known compound **14** was carried out according to a reference procedure.[1] A mixture of 2-iodoaniline (**12**, 249 mg, 1.1 mmol), 1-(triisopropylsilyl)-1*H*-pyrrole-3-boronic acid pinacol ester (**13**, 500 mg, 1.4 mmol), K_3PO_4 (485 mg, 2.3 mmol), $Pd(OAc)_2$ (9 mg, 0.04 mmol), and SPhos (33 mg, 0.08 mmol) in *n*-BuOH/H_2O (4 mL:2 mL) was stirred at 80 °C for 2 h under a N_2 atmosphere. The reaction mixture was quenched with water, and then extracted with EtOAc. The organic layer was washed with water and brine, dried over Na_2SO_4, and evaporated *in vacuo*. The residue was purified by column chromatography using EtOAc/hexane 1:5 (*v*/*v*) as an eluent to give the 2-(pyrrol-3-yl)aniline **14** (248 mg, 84%). Yellow oil; ^{1}H NMR (300 MHz, $CDCl_3$) δ 7.25 (dd, J = 7.5 and 1.7 Hz, 1H), 7.05 (td, J = 7.5 and 1.7 Hz, 1H), 6.95 (dd, J = 2.2 and 1.5

Hz, 1H), 6.84 (dd, $J = 2.8$ and 2.2 Hz, 1H), 6.79 (td, $J = 7.5$ and 1.7 Hz, 1H), 6.74 (dd, $J = 7.5$ and 1.7 Hz, 1H), 6.51 (dd, $J = 2.8$ and 1.5 Hz, 1H), 3.93 (br s, 2H), 1.47 (sept, $J = 7.3$ Hz, 3H), 1.13 (d, $J = 7.3$ Hz, 18H) ppm; ^{13}C NMR (75 MHz, $CDCl_3$) δ 143.6, 129.7, 126.9, 124.7, 123.8, 122.6, 122.3, 118.6, 115.5, 110.7, 17.8, 11.7 ppm; HRMS (EI) *m/z*: [M^+] calcd for $C_{19}H_{30}N_2Si$, 314.2178; found, 314.2180. IR (ATR) ν (cm^{-1}): 3290.
The spectral data were in agreement with the literature values.[1]

2-(1*H*-Pyrrol-3-yl)aniline (15)[2]

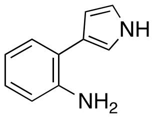

The synthesis of the known compound **15** was performed by a method[3] different to the procedure described in ref. 2. A solution of TBAF (1.0 M in THF, 0.21 mL, 0.21 mmol) was added dropwise to a solution of TIPS-protected 2-(pyrrol-3-yl)aniline **14** (56 mg, 0.178 mmol) in THF (5 mL) at 0 °C under a N_2 atmosphere. After stirring at rt for 10 min, the reaction was quenched with water, and then extracted with EtOAc. The organic layer was washed with brine, dried over Na_2SO_4, and evaporated *in vacuo.* The residue was purified by column chromatography using EtOAc/hexane 1:4 (*v/v*) as an eluent to give the 2-(1*H*-pyrrol-3-yl)aniline (**15**, 65 mg, 65%). Yellow oil; ^{1}H NMR (300 MHz, $CDCl_3$) δ 8.35 (br s, 1H) , 7.24 (dd, $J = 7.5$ and 1.7 Hz, 1H), 7.07 (td, $J = 7.5$ and 1.7 Hz, 1H) , 6.98–7.00 (m, 1H), 6.87–6.89 (m, 1H) , 6.79 (td, $J = 7.5$ and 1.7 Hz, 1H), 6.75 (dd, $J = 7.5$ and 1.7 Hz, 1H) , 6.44–6.46 (m, 1H), 3.94 (br s, 2H) ppm; ^{13}C NMR (75 MHz, $CDCl_3$) δ 143.8, 129.9, 127.2, 122.2, 121.9, 118.6, 118.4, 116.1, 115.5, 108.7 ppm; HRMS (EI) *m/z*: [M^+] calcd for $C_{10}H_{10}N_2$, 158.0844; found, 158.0848; IR (ATR) ν (cm^{-1}): 3406.
The spectral data were in agreement with the literature values.[2]

_N_-Phenyl-_N'_-[2-(1_H_-pyrrol-3-yl)phenyl]urea (16a)

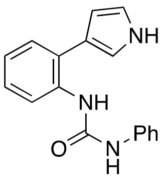

A solution of phenyl isocyanate (62 µL, 0.57 mmol) in CH_2Cl_2 (1 mL) was added dropwise to a solution of aniline **14** (60 mg, 0.38 mmol) in CH_2Cl_2 (2 mL) at rt under a N_2 atmosphere. After stirring at rt for 12 h, the reaction mixture was evaporated *in vacuo*. The residue was purified by column chromatography using EtOAc/hexane 3:7 (v/v) as an eluent to give the urea **16a** (57 mg, 54%). White solid; mp = 160–161 °C; ^{1}H NMR (300 MHz, DMSO-d_6) δ 11.09 (br s, 1H), 9.17 (s, 1H), 7.81 (dd, J = 7.8 and 1.7 Hz, 1H), 7.75 (br s, 1H), 7.44 (dd, J = 7.8 and 1.7 Hz, 2H), 7.23–7.32 (m, 3H), 7.14 (td, J = 7.8 and 1.7 Hz, 1H), 6.99–7.05 (m, 2H), 6.89–6.95 (m, 2H), 6.27–6.30 (m, 1H) ppm; ^{13}C NMR (75 MHz, DMSO-d_6) δ 152.9, 140.1, 135.1 129.2, 128.8, 127.9, 125.7, 123.0, 122.4, 121.6, 119.5, 118.7, 117.9, 116.7, 107.9 ppm; HRMS (EI) *m/z*: [M$^+$] calcd for $C_{17}H_{15}N_3O$, 277.1215; found, 277.1227; IR (ATR) ν(cm^{-1}): 3325, 3286,1631; IR (ATR) ν(cm^{-1}): 3302, 1651.

_N_-Phenyl-_N'_-[2-(1-(triisopropylsilyl)-1_H_-pyrrol-3-yl)phenyl]urea (16b)

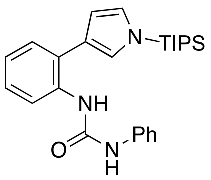

The same procedure as above was carried out using aniline **15** (300 mg, 0.96 mmol) to give the urea **16b** (220 mg, 53%). White solid; mp = 199–201 °C; ^{1}H NMR (300 MHz, DMSO-d_6) δ 8.96 (br s, 1H), 7.74 (br s, 1H), 7.61 (dd, J = 7.4 and 1.7 Hz, 1H), 7.38–7.44 (m, 3H), 7.23–7.28 (m, 2H), 7.17 (td, J = 7.4 and 1.7 Hz, 1H), 7.07–7.12 (m, 2H), 6.91–6.96 (m, 2H), 6.51 (dd, J = 2.8 and 1.5 Hz, 1H), 1.48 (sept, J = 7.4 Hz, 3H), 1.05 (d, J = 7.4 Hz, 18H) ppm; ^{13}C NMR (75 MHz, DMSO-d_6) δ 153.2, 140.1, 134.5, 129.2, 129.0, 128.7, 125.8, 124.90, 124.85, 124.1, 122.9, 122.6, 121.5, 117.8, 110.6, 17.6, 10.9 ppm; HRMS (EI) *m/z*: [M$^+$] calcd for $C_{26}H_{35}N_3OSi$, 433.2549; found, 433.2553; IR (ATR) ν(cm^{-1}): 3325, 1651.

N-Phenyl-N'-[2-(1-triisopropylsilyl-1H-pyrrol-3-yl)phenyl]carbodiimide (17b)

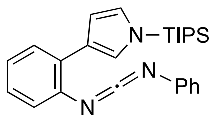

A solution of CBr_4 (380 mg, 1.2 mmol) in CH_2Cl_2 (2 mL) was added dropwise to a solution of urea **16b** (100 mg, 0.23 mmol), PPh_3 (303 mg, 1.2 mmol), and Et_3N (0.32 mL, 2.3 mmol) in CH_2Cl_2 (8 mL) at 0 °C. After stirring at rt for 2 h, the reaction mixture was then evaporated *in vacuo*. The residue was washed with EtOAc/hexane 1:4 (v/v) for 3 times and the filtrate was evaporated *in vacuo* to give the carbodiimide **17b** (56 mg, 58%). Yellow oil; ^{1}H NMR (300 MHz, $CDCl_3$) δ 7.64–7.70 (m, 1H), 7.51–7.55 (m, 1H), 7.46–7.49 (m, 1H), 7.34 (dd, J = 2.2 and 1.5 Hz, 1H), 7.26–7.35 (m, 2H), 7.11–7.17 (m, 4H), 6.80 (dd, J = 2.8 and 2.2 Hz, 1H), 6.71 (dd, J = 2.8 and 1.5 Hz, 1H), 1.46 (sept, J = 7.4 Hz, 3H), 1.12 (d, J = 7.4 Hz, 18H) ppm; ^{13}C NMR (75 MHz, $CDCl_3$) δ 134.1, 132.1, 130.7, 129.4, 128.8, 128.6, 128.5, 126.2, 125.9, 125.7, 125.0, 124.3, 124.0, 123.0, 110.6, 17.8, 11.7 ppm; HRMS (EI) *m/z*: [M^+] calcd for $C_{26}H_{33}N_3Si$, 415.2444; found, 415.2448; IR (ATR) ν (cm^{-1}): 2133.

N-Phenyl-3H-pyrrolo[2,3-c]quinolin-4-amine (18)

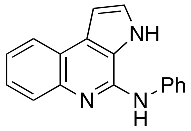

A solution of TBAF (1.0 M in THF, 80 μL, 0.080 mmol) was added dropwise to a solution of carbodiimide **17b** (28 mg, 0.067 mmol) in 1,2-dichlorobenzene (2.0 mL) under a N_2 atmosphere. After stirring at 80 °C for 1 h, the reaction mixture was evaporated *in vacuo*. The residue was purified by column chromatography using EtOAc/hexane 1:5 (v/v) as an eluent to give the pyrroloquinoline **18** (10 mg, 58%). Yellow oil; ^{1}H NMR (300 MHz, $CDCl_3$) δ 8.05 (dd, J = 7.8 and 1.5 Hz, 1H), 7.79 (d, J = 7.8 Hz, 1H), 7.46 (td, J = 7.8 and 1.5 Hz, 1H), 7.37 (td, J = 7.8 and 1.5 Hz, 1H), 7.28–7.35 (m, 4H), 7.18 (d, J = 2.9 Hz, 1H), 7.05–7.09 (m, 1H), 6.95 (d, J = 2.9 Hz, 1H) ppm; ^{13}C NMR (75 MHz, $CDCl_3$) δ 143.3, 141.2, 140.9, 129.8, 129.4, 126.5, 125.3, 125.1, 123.7, 123.2, 122.7, 121.5, 121.1, 120.1, 102.1 ppm; HRMS (EI) *m/z*: [M^+] calcd for $C_{17}H_{13}N_3$, 259.1109; found, 259.1117; IR (ATR) ν (cm^{-1}): 3332.

N-Benzyl-N'-[2-(1-triisopropylsilyl-1H-pyrrol-3-yl)phenyl]urea (19a)

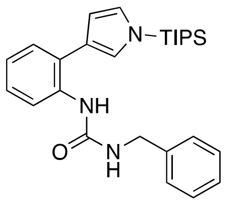

A solution of benzyl isocyanate (0.29 mL, 2.4 mmol) in CH_2Cl_2 (5 mL) was added dropwise to a solution of 2-(1*H*-pyrrol-3-yl)aniline **14** (500 mg, 1.6 mmol) in CH_2Cl_2 (10 mL) at rt under N_2 atmosphere. After stirring at rt for 12 h, the reaction mixture was evaporated *in vacuo*. The residue was purified by column chromatography using EtOAc/hexane 1:4 (v/v) as an eluent to give the urea **19a** (355 mg, 50%). White solid; mp = 136–138 °C; ^{1}H NMR (300 MHz, $CDCl_3$) δ 7.74 (dd, *J* = 7.5 and 1.4 Hz, 1H), 7.39 (dd, *J* = 7.5 and 1.4 Hz, 1H), 7.26–7.32 (m, 3H), 7.19–7.24 (m, 3H), 7.12 (td, *J* = 7.5 and 1.4 Hz, 1H), 6.96 (dd, *J* = 2.0 and 1.6 Hz, 1H), 6.84 (dd, *J* = 2.8 and 2.0 Hz, 1H), 6.46–6.47 (m, 2H), 4.84 (t, *J* = 6.1 Hz, 1H), 4.42 (d, *J* = 6.1 Hz, 1H), 1.45 (sept, *J* = 7.4 Hz, 3H), 1.11 (d, *J* = 7.4 Hz, 18H) ppm; ^{13}C NMR (75 MHz, $CDCl_3$) δ 155.8, 139.0, 134.7, 129.1, 129.6, 128.6, 127.5, 127.3, 126.9, 125.2, 124.5, 123.3, 123.0, 122.7, 110.6, 44.3, 17.8, 11.6 ppm; HRMS (EI) *m/z*: [M^+] calcd for $C_{27}H_{37}N_3OSi$, 447.2706; found, 447.2714. IR (ATR) ν (cm^{-1}): 3290, 1624.

N-(2-Bromophenyl)-N'-[2-(1-triisopropylsilyl-1H-pyrrol-3-yl)phenyl]urea (19b)

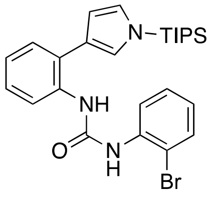

The analogous procedure as described above was carried out using aniline **14** (347 mg, 1.11 mmol) and the corresponding aryl isocyanate to give the urea **19b** (526 mg, 98%). White solid; mp = 99–100 °C; ^{1}H NMR (300 MHz, $CDCl_3$) δ 8.17 (dd, *J* = 7.8 and 1.5 Hz, 1H), 7.77 (dd, *J* = 7.8 and 1.5 Hz, 1H), 7.43–7.48 (m, 2H), 7.28–7.31 (m, 1H), 7.20 (td, *J* = 7.8 and 1.5 Hz, 1H), 6.97–6.99 (m, 2H), 6.91 (td, *J* = 7.8 and 1.5 Hz, 1H), 6.83 (dd, *J* = 2.8 and 2.2 Hz, 1H), 6.68 (br s, 1H), 6.49 (dd, *J* = 2.8 and 1.5 Hz, 1H), 1.45 (sept, *J* = 7.5 Hz, 3H), 1.11 (d, *J* = 7.5 Hz, 18H) ppm; ^{13}C NMR (75 MHz, $CDCl_3$) δ 152.9,

136.3, 133.7, 132.5, 132.1, 130.1, 129.8 128.2, 127.0, 125.5, 125.3, 124.2, 124.1, 123.1, 122.5, 121.5, 113.5, 110.7, 17.8, 11.6 ppm; HRMS (EI) *m/z*: [M$^+$] calcd for $C_{26}H_{34}BrN_3OSi$, 511.1655; found, 511.1665. IR (ATR) ν(cm^{-1}): 3294, 1655.

N-[3-(2-Bromophenyl)propyl]-N'-[2-(1-triisopropylsilyl-1H-pyrrol-3-yl)phenyl]urea (19c)

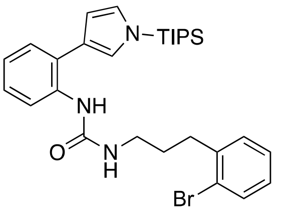

To a solution of 4-(2-bromophenyl)butanoic acid (300 g, 1.2 mmol) in toluene (15 mL) was added diphenylphosporyl azide (0.3 mL, 1.6 mmol) and Et_3N (0.25 mL, 1.8 mmol). After stirring at rt for 30 min, the reaction mixture was evaporated *in vacuo* to give the isocyanate as a yellow oil. The isocyanate was used in the next reaction without purification. A solution of the isocyanate in CH_2Cl_2 (3.0 mL) was added dropwise to a solution of aniline **14** (193 mg, 0.62 mmol) in CH_2Cl_2 (3.0 mL) at rt under N_2 atmosphere. After stirring at rt for 20 h, the reaction mixture was evaporated *in vacuo*. The residue was purified by column chromatography using EtOAc/hexane 1:4 (v/v) as an eluent to give the urea **19c** (215 mg, 64%). White solid; mp = 134–135 °C; ^{1}H NMR (300 MHz, $CDCl_3$) δ 7.65 (dd, J = 8.0 and 1.1 Hz, 1H), 7.48 (dd, J = 8.0 and 1.1 Hz, 1H), 7.42 (dd, J = 8.0 and 1.1 Hz, 1H), 7.16–7.26 (m, 3H), 7.14 (td, J = 8.0 and 2.2 Hz, 1H), 7.00–7.06 (m, 1H), 6.96 (dd, J = 2.9 and 1.5 Hz, 1H), 6.83 (dd, J = 2.9 and 2.0 Hz, 1H), 6.49 (dd, J = 2.9 and 1.5 Hz, 1H), 6.33 (br s, 1H), 4.64 (t, J = 6.0 Hz, 1H), 3.25 (dt J = 7.2 and 6.0 Hz, 2H), 2.72 (t, J = 7.2 Hz, 2H), 1.78 (quin, J = 7.2 Hz, 2H), 1.47 (sept, J = 7.5 Hz, 3H), 1.11 (d, J = 7.5 Hz, 18H) ppm; ^{13}C NMR (75 MHz, $CDCl_3$) δ 156.1, 140.8, 134.7, 132.7, 130.3, 129.62, 129.57, 127.6, 127.5, 126.8, 125.1, 124.7, 124.3, 123.9, 123.0, 122.7, 110.7, 39.7, 33.3, 30.2, 17.8, 11.6 ppm; HRMS (EI) *m/z*: [M$^+$] calcd for $C_{29}H_{40}BrN_3OSi$, 553.2124; found, 553.2128. IR (ATR) ν(cm^{-1}): 3294, 1624.

***N*-[3-(2-Bromophenyl)-3-oxopropyl]-*N'*-[2-(1-triisopropylsilyl-1*H*-pyrrol-3-yl)phenyl]urea (19d)**

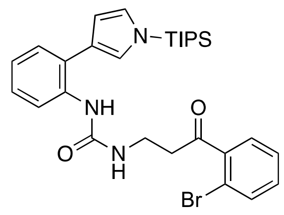

The same procedure as above was carried out using aniline **14** (73 mg, 0.23 mmol) and 4-(2-bromophenyl)-4-oxobutanoic acid (120 mg, 0.47 mmol) to give the urea **19d** (62 mg, 98%). White solid; mp = 121–123 °C; ^{1}H NMR (300 MHz, $CDCl_3$) δ 7.73 (dd, *J* = 8.0 and 1.2 Hz, 1H), 7.59 (dd, *J* = 8.0 and 1.2 Hz, 1H), 7.35–7.39 (m, 3H), 7.29–7.33 (m, 1H), 7.23 (td, *J* = 8.0 and 1.2 Hz, 1H), 7.11 (td, *J* = 8.0 and 1.2 Hz, 1H), 6.92 (dd, *J* = 2.2 and 1.5 Hz, 1H), 6.84 (dd, *J* = 2.8 and 2.2 Hz, 1H), 6.45 (dd, *J* = 2.8 and 1.5 Hz, 1H), 6.42 (br s, 1H), 5.15 (t, *J* = 6.5 Hz, 1H), 3.63 (dt *J* = 6.5 and 6.1 Hz, 2H), 3.18 (t, *J* = 6.1 Hz, 2H), 1.47 (sept, *J* = 7.5 Hz, 3H), 1.12 (d, *J* = 7.5 Hz, 18H) ppm; ^{13}C NMR (75 MHz, $CDCl_3$) δ 203.4, 155.8, 140.8, 134.9, 133.9, 131.8, 129.7, 128.7, 128.5, 127.5, 126.9, 125.3, 124.3, 123.0, 122.8, 122.7, 118.7, 110.7, 42.8, 35.2, 17.8, 11.6 ppm; HRMS (EI) *m/z*: [M^+] calcd for $C_{29}H_{38}BrN_3O_2Si$, 567.1917; found, 567.1923; IR (ATR) ν(cm^{-1}): 3317, 1705, 1651.

***N*-Benzyl-*N'*-[2-(1-triisopropylsilyl-1*H*-pyrrol-3-yl)phenyl]carbodiimide (20a)**

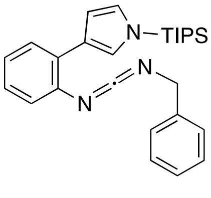

A solution of CBr_4 (148 mg, 0.45 mmol) in CH_2Cl_2 (2 mL) was added dropwise to a solution of urea **19a** (100 mg, 0.22 mmol), PPh_3 (117 mg, 0.45 mmol), and Et_3N (0.12 mL, 0.90 mmol) in CH_2Cl_2 (8 mL) at 0 °C. After stirring at rt for 2 h, the reaction mixture was evaporated *in vacuo*. The residue was purified by column chromatography using EtOAc/hexane 1:9 (v/v) as an eluent to give the carbodiimide **20a** (62 mg, 64%). Yellow oil; ^{1}H NMR (300 MHz, $CDCl_3$) δ 7.45–7.48 (m, 1H), 7.34–7.36 (m, 4H), 7.28–7.30 (m, 2H), 7.03–7.11 (m, 3H), 6.79 (dd, *J* = 2.8 and 2.1 Hz, 1H), 6.66 (dd, *J* = 2.8 and 1.5 Hz, 1H), 4.52 (s, 2H), 1.47 (sept, *J* = 7.3 Hz, 3H), 1.12 (d, *J* = 7.3 Hz, 18H) ppm; ^{13}C NMR

(75 MHz, $CDCl_3$) δ 138.2, 136.8, 135.8, 130.3, 128.7, 127.6, 127.5, 127.3, 126.0, 125.2, 125.0, 124.1, 123.2, 110.6, 50.4, 17.9, 11.7 ppm; HRMS (EI) *m/z*: [M^+] calcd for $C_{27}H_{35}N_3Si$, 429.2600; found, 429.2610. IR (ATR) ν (cm^{-1}): 2137.

_N_-(2-Bromophenyl)-_N'_-[2-(1-triisopropylsilyl-1_H_-pyrrol-3-yl)phenyl]carbodiimide (20b)

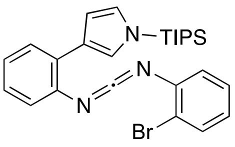

A solution of CBr_4 (119 mg, 0.361 mmol) in CH_2Cl_2 (1 mL) was added dropwise to a solution of urea **19b** (35 mg, 0.072 mmol), PPh_3 (94 mg, 0.36 mmol), and Et_3N (0.1 mL, 0.72 mmol) in CH_2Cl_2 (2 mL) at 0 °C. After stirring at rt for 2 h, the reaction mixture was evaporated *in vacuo* to give the crude carbodiimide **20b**. The carbodiimide **20b** was used in the next reaction without purification, because it was unstable.

_N_-(2-Bromophenyl)-3_H_-pyrrolo[2,3-_c_]quinolin-4-amine (22b)

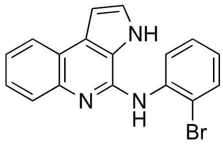

A solution of TBAF (1.0 M in THF, 86 μL, 0.0864 mmol) was added dropwise to a solution of the crude carbodiimide **20b** in 1,2-dichlorobenzene (3 mL) under a N_2 atmosphere. After stirring at 80 °C for 1 h, the reaction mixture was evaporated *in vacuo*. The residue was purified by column chromatography using EtOAc/hexane 3:7 (v/v) as an eluent to give the pyrroloquinolin **22b** (12 mg, 49%). Colorless oil; ^1H NMR (300 MHz, DMSO-d_6) δ 11.99 (br s, 1H), 8.33 (br s, 1H), 8.03–8.13 (m, 2H), 7.70 (d, J = 8.0 Hz, 1H), 7.60–7.63 (m, 1H), 7.57 (d, J = 8.0 Hz, 1H), 7.29–7.46 (m, 3H), 7.03–713 (m, 2H) ppm; ^{13}C NMR (75 MHz, DMSO-d_6) δ 142.9, 141.6, 138.1, 132.7, 128.4, 127.8, 126.8, 126.3, 126.0, 125.5, 124.9, 122.54, 122.46, 121.3, 119.4, 116.5, 101.6 ppm; HRMS (EI) *m/z*: [M^+] calcd for $C_{17}H_{12}BrN_3$, 337.0215; found, 337.0225. IR (ATR) ν (cm^{-1}): 2962.

N-[3-(2-Bromophenyl)propyl]-N'-[2-(1-triisopropylsilyl-1H-pyrrol-3-yl)phenyl] carbodiimide (20c)

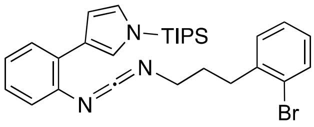

A solution of CBr_4 (1.70 g, 5.1 mmol) in CH_2Cl_2 (10 mL) was added dropwise to a solution of urea **19c** (568 mg, 1.03 mmol), PPh_3 (1.34 g, 5.1 mmol), and Et_3N (1.4 mL, 10.3 mmol) in CH_2Cl_2 (20 mL) at 0 °C. After stirring at rt for 2 h, the reaction mixture was evaporated *in vacuo*. The residue was purified by column chromatography using EtOAc/hexane 1:4 (v/v) as an eluent to give the carbodiimide **20c** (411 mg, 75%). Yellow oil; ^{1}H NMR (300 MHz, $CDCl_3$) δ 7.48–7.54 (m, 2H), 7.34 (dd, J = 2.0 and 1.5 Hz, 1H), 7.20–7.23 (m, 3H), 7.03–7.14 (m, 3H), 6.79 (dd, J = 2.7 and 2.0 Hz, 1H), 6.70 (dd, J = 2.7 and 1.5 Hz, 1H), 3.41 (t, J = 6.6 Hz, 2H), 2.86 (dt J = 6.6 and 6.1 Hz, 2H), 1.95 (t, J = 6.1 Hz, 2H), 1.47 (sept, J = 7.5 Hz, 3H), 1.13 (d, J = 7.5 Hz, 18H) ppm; ^{13}C NMR (75 MHz, $CDCl_3$) δ 140.5, 136.3, 135.7, 132.9, 130.5, 130.2, 128.7, 127.8, 127.5, 126.0, 125.0, 124.8, 124.4, 124.0, 123.3, 110.6, 46.1, 33.4, 31.3, 17.8, 11.7 ppm; HRMS (EI) *m/z*: [M$^+$] calcd for $C_{29}H_{38}BrN_3Si$, 535.2018; found, 535.2012. IR (ATR) ν (cm^{-1}): 2137.

N-[3-(2-Bromophenyl)-3-oxopropyl]-N'-[2-(1-triisopropylsilyl-1H-pyrrol-3-yl)phenyl]carbodiimide (20d)

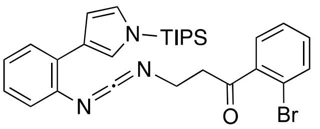

A solution of CBr_4 (443 mg, mmol) in CH_2Cl_2 (5 mL) was added dropwise to a solution of urea **19d** (152 mg, 0.27mmol), PPh_3 (351 mg, 1.34 mmol), and Et_3N (0.4 mL, 2.68 mmol) in CH_2Cl_2 (4 mL) at 0 °C. After stirring at rt for 2 h, the reaction mixture was evaporated *in vacuo*. The residue was purified by column chromatography using EtOAc/hexane 1:9 (v/v) as an eluent to give the carbodiimide **20d** (99 mg, 68%). Brown oil; ^{1}H NMR (300 MHz, $CDCl_3$) δ 7.58–7.61 (m, 1H), 7.45–7.49 (m, 1H), 7.36–7.41 (m, 1H), 7.28–7.32 (m, 3H), 7.17–7.21 (m, 1H), 7.07–7.13 (m, 2H), 6.79 (dd, J = 2.8 and 2.2 Hz, 1H), 6.68 (dd, J = 2.8 and 1.5 Hz, 1H), 3.79 (t, J = 6.8 Hz, 2H), 3.26 (t J = 6.8 Hz, 2H), 1.45 (sept, J = 7.6 Hz, 3H), 1.12 (d, J = 7.6 Hz, 18H) ppm; ^{13}C NMR (75 MHz, $CDCl_3$) δ 201.4, 141.1, 136.1, 135.7, 133.7, 131.8, 130.3, 128.8, 128.7,

127.5, 126.1, 125.3, 125.0, 124.0, 123.2, 118.7, 110.6, 43.8, 41.7, 17.8, 11.7 ppm; HRMS (EI) *m/z*: [M$^+$] calcd for $C_{29}H_{35}BrN_3OSi$, 549.1811; found, 549.1823. IR (ATR) ν(cm^{-1}): 2133.

N-Benzyl-3*H*-pyrrolo[2,3-*c*]quinolin-4-amine (22a)

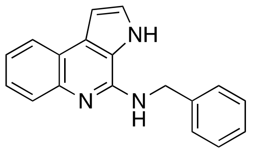

A solution of TBAF (1.0 M in THF, 0.14 mL, 0.14 mmol) was added dropwise to a solution of carbodiimide **19a** (50 mg, 0.12 mmol) in 1,2-dichlorobenzene (10 mL) under N_2 atmosphere. After stirring at 80 °C for 1 h, the reaction mixture was evaporated *in vacuo*. The residue was purified by column chromatography using EtOAc/hexane 1:9 (v/v) as an eluent to give the pyrroloquinolin **22a** (26 mg, 90%). Brown solid; mp = 145–146 °C; ^{1}H NMR (300 MHz, $CDCl_3$) δ 7.95 (d, *J* = 7.7 Hz, 1H), 7.77 (d, *J* = 7.7 Hz, 1H), 7.37 (t, *J* = 7.7 Hz, 1H), 7.29 (t, *J* = 7.7 Hz, 1H), 7.06–7.19 (m, 6H), 6.86 (d, *J* = 2.8 Hz, 1H), 4.72 (s, 2H) ppm; ^{13}C NMR (75 MHz, $CDCl_3$) δ 145.9, 142.3, 138.5, 128.8, 128.5, 127.9, 127.3, 126.3, 125.4, 124.8, 122.7, 122.6, 120.5, 119.1, 102.4, 45.6 ppm; HRMS (EI) *m/z*: [M$^+$] calcd for $C_{18}H_{15}N_3$, 273.1266; found, 273.1276. IR (ATR) ν(cm^{-1}): 3329.

N-[3-(2-bromophenyl)propyl]-3*H*-pyrrolo[2,3-*c*]quinolin-4-amine (22c)

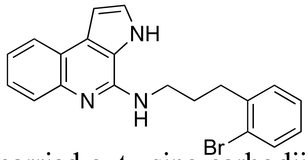

The same procedure as above was carried out using carbodiimide **19c** (102 mg, 0.1 mmol) to give the pyrrolo[2,3-*c*]quinoline **22c** (45 mg, 61%). White solid; mp = 158–159 °C; ^{1}H NMR (300 MHz, $CDCl_3$) δ ^{1}H NMR (300 MHz, $CDCl_3$) δ 7.93 (d, *J* = 7.8 Hz, 1H), 7.86 (d, *J* = 8.3 Hz, 1H), 7.40 (d, *J* = 7.8 Hz, 1H), 7.37 (dd, *J* = 8.3 Hz, 1H), 7.33 (d, *J* = 2.8 Hz, 1H), 7.22–7.27 (m, 1H), 7.09 (t, *J* = 7.8 Hz, 1H), 6.98 (t, *J* = 7.8 Hz, 1H), 6.94 (d, *J* = 2.8 Hz, 1H), 6.74 (d, *J* = 7.8 Hz, 1H), 5.20 (br s, 1H), 3.30 (t, *J* = 7.2 Hz, 2H), 2.29 (t, *J* = 7.2 Hz, 2H), 1.28 (quin, *J* = 7.1 Hz, 2H) ppm; ^{13}C NMR (75 MHz, $CDCl_3$) δ 147.3, 142.6, 140.5, 132.5, 130.1, 128.7, 127.5, 127.3, 126.3, 125.1, 124.8, 124.3, 122.8, 122.4, 120.9, 119.7, 102.5, 41.1, 33.0, 28.7 ppm; HRMS (EI) *m/z*: [M$^+$] calcd for $C_{20}H_{18}BrN_3$, 379.0684; found, 379.0692. IR (ATR) ν(cm^{-1}): 3309.

4-[1,2,3,4-Tetrahydroquinolin-1-yl)]-3*H*-pyrrolo[2,3-*c*]quinoline (23)

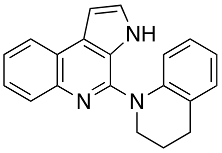

A mixture of pyrrolo[2,3-*c*]quinoline **22c** (38 mg, 0.10 mmol), $Pd(OAc)_2$ (1.12 mg, 5.0 μmol), $Cu(OAc)_2$ (9.1 mg, 50 μmol) and K_2CO_3 (69 mg, 0.50 mmol) in toluene (2.0 mL) was refluxed for 3 h under N_2 atmosphere. The reaction mixture was filtered through Celite pad and the organic layer was evaporated *in vacuo*. The residue was purified by column chromatography using EtOAc/hexane 1:4 (v/v) as an eluent to give the tetrahydroquinoline **23** (10 mg, 34%). Yellow oil; ^{1}H NMR (300 MHz, $CDCl_3$) δ 8.12 (dd, J = 7.8 and 1.6 Hz, 1H), 8.00 (dd, J = 7.8 and 1.6 Hz, 1H), 7.91 (br s, 1H), 7.54 (td, J = 7.8 and 1.6 Hz, 1H), 7.45 (td, J = 7.8 and 1.6 Hz, 1H), 7.22 (dd, J = 7.8 and 1.6 Hz, 1H), 7.14 (t, J = 2.8 Hz, 1H), 7.03 (td, J = 8.0 and 1.6 Hz, 1H), 6.93–6.98 (m, 2H), 6.75 (dd, J = 7.8 and 1.6 Hz, 1H), 4.19 (t, J = 6.2 Hz, 2H), 2.92 (t, J = 6.2 Hz, 2H), 1.12 (quin, J = 6.2 Hz, 2H) ppm; ^{13}C NMR (75 MHz, $CDCl_3$) δ 145.1, 142.8, 141.2, 130.1, 129.2, 129.1, 127.6, 126.3, 126.2, 124.4, 123.9, 122.5, 122.0, 121.9, 121.7, 118.7, 101.6, 47.3, 27.5, 24.1 ppm; HRMS (EI) *m/z*: [M^+] calcd for $C_{20}H_{17}N_3$, 299.1422; found, 299.1432.

5-Methoxy-2-(1-triisopropylsilyl-*1H*-pyrrol-3-yl)aniline (25)

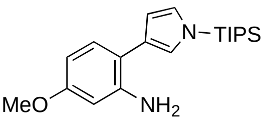

The synthesis of compound **25** was carried out according to the reference procedure.[1] A mixture of 2-iodo-5-methoxyaniline (**24**, 249 mg, 1.1 mmol), 1-(triisopropylsilyl)-1*H*-pyrrole-3-boronic acid pinacol ester (**13**, 500 mg, 1.4 mmol), K_3PO_4 (486 mg, 2.3 mmol), $Pd(OAc)_2$ (13 mg, 57 μmol), and SPhos (47 mg, 114 μmol) in *n*-BuOH/H_2O (6 mL:3 mL) was stirred at 80 °C for 2 h under N_2 atmosphere. The reaction was quenched with water, and then the mixture extracted with EtOAc. The EtOAc layer was washed with water and brine, dried over Na_2SO_4, and evaporated *in vacuo*. The residue was purified by column chromatography using EtOAc/hexane 1:4 (*v*/*v*) to give the 2-(pyrrol-3-yl)aniline **25** (227 mg, 66%). Brown oil; ^{1}H NMR (300 MHz, $CDCl_3$) δ 7.17 (d, J = 8.3 Hz, 1H), 6.87-6.89

(m, 1H), 6.83 (t, J = 2.5 Hz, 1H), 6.44-6.46 (m, 1H), 6.35–6.40 (m, 2H), 3.78 (s, 3H), 1.47 (sept, J = 7.6 Hz, 3H), 1.12 (d, J = 7.6 Hz, 18H) ppm; ^{13}C NMR (75 MHz, $CDCl_3$) δ 159.0, 144.6, 130.5, 124.6, 123.4, 121.9, 115.8, 110.7, 104.0, 101.0, 55.1, 17.8, 11.6 ppm; HRMS (EI) *m/z*: [M^+] calcd for $C_{19}H_{30}N_2Si$, 314.2178; found, 314.2184; IR (ATR) ν (cm^{-1}): 2943.

***N*-[3-(2-Bromophenyl)propyl]-*N'*-[5-methoxy-2-(1-triisopropylsilyl-1*H*-pyrrol-3-yl)phenyl]urea (27a)**

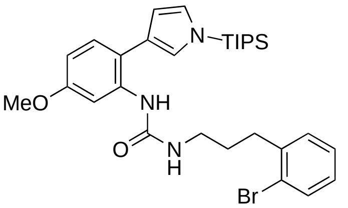

To a solution of 4-(2-bromophenyl)butanoic acid (620 mg, 2.6 mmol) in toluene (15 mL) was added diphenylphosporyl azide (0.70 mL, 3.3 mmol) and Et_3N (0.50 mL, 3.4 mmol). After stirring at rt for 30 min, the reaction mixture was evaporated *in vacuo* to give the isocyanate as yellow oil. The isocyanate was used in the next reaction without purification. A solution of the isocyanate in CH_2Cl_2 (11 mL) was added dropwise to a solution of the aniline **25** (438 mg, 1.3 mmol) in CH_2Cl_2 (10 mL) at rt under a N_2 atmosphere. After stirring at rt for 20 h, the reaction mixture was evaporated *in vacuo*. The residue was purified by column chromatography using EtOAc/hexane 1:4 (v/v) as an eluent to give the urea **27a** (448 mg, 60%). White solid; mp = 117–118 °C; ^{1}H NMR (300 MHz, $CDCl_3$) δ 7.49 (d, J = 8.3 Hz, 1H), 7.45 (d, J = 2.6 Hz, 1H), 7.27 (d, J = 8.3 Hz, 1H), 7.17–7.21 (m, 2H), 7.00–7.06 (m, 1H), 6.86 (t, J = 1.5 Hz, 1H), 6.82 (t, J = 2.6 Hz, 1H), 6.68 (dd, J = 8.3 and 2.6 Hz, 1H), 6.55 (br s, 1H), 6.41 (dd, J = 2.6 and 1.5 Hz, 1H), 4.69 (br s, 1H), 3.83 (s, 3H), 3.22–3.26 (m, 2H), 2.73 (t, J = 7.5 Hz, 2H), 1.78 (quin, J = 7.5 Hz, 2H) 1.46 (sept, J = 7.4 Hz, 3H), 1.11 (d, J = 7.4 Hz, 18H) ppm; ^{13}C NMR (75 MHz, $CDCl_3$) δ 158.6, 155.7, 140.7, 136.1, 132.7, 130.4, 130.3, 127.7, 127.5, 125.1, 124.3, 122.6, 122.4, 120.9, 110.6, 110.3, 107.5, 55.4, 39.8, 33.3, 30.2, 17.8, 11.6 ppm; HRMS (EI) *m/z*: [M^+] calcd for $C_{30}H_{42}BrN_3O_2Si$, 583.2230; found, 583.2237. IR (ATR) ν (cm^{-1}): 3298, 1631.

N-[3-(2-Iodophenyl)propyl]-N'-[5-methoxy-2-(1-triisopropylsilyl-1H-pyrrol-3-yl)phenyl]urea (27b)

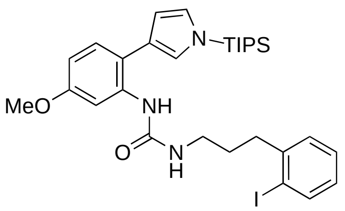

The same procedure as above was carried out using aniline **25** (50 mg, 0.145 mmol) and 4-(2-bromophenyl)butanoic acid (105 mg, 0.362 mmol) to give the urea **27b** (66 mg, 70%). White solid; mp = 124–125 °C; ^{1}H NMR (300 MHz, $CDCl_3$) δ 7.77 (dd, J = 7.8 and 1.2 Hz, 1H), 7.46 (d, J = 2.8 Hz, 1H), 7.26–7.28 (m, 1H), 7.20 (td, J = 7.8 and 1.2 Hz, 1H), 6.82–6.89 (m, 3H), 6.68 (dd, J = 8.3 and 2.8 Hz, 1H), 6.48 (br s, 1H), 6.42 (dd, J = 2.5 and 1.3 Hz, 1H), 4.65 (t, J = 5.8 Hz, 1H), 3.83 (s, 3H), 3.27 (dt, J = 7.9 and 5.8 Hz, 2H), 2.72 (t, J = 7.9 Hz, 1H), 1.76 (quin, J = 7.9 Hz, 2H) 1.46 (sept, J = 7.8 Hz, 3H), 1.11 (d, J = 7.8 Hz, 18H) ppm; ^{13}C NMR (75 MHz, $CDCl_3$) δ 158.7, 155.7, 144.0, 139.4, 136.1, 130.4, 129.4, 128.4, 127.8, 125.1, 122.6, 122.4, 121.0, 110.7, 110.3, 107.6, 100.5, 55.4, 39.8, 38.0, 30.6, 17.8, 11.6 ppm; HRMS (EI) m/z: [M^+] calcd for $C_{30}H_{42}IN_3O_2Si$, 631.2091; found, 631.2097. IR (ATR) ν(cm^{-1}): 3275, 2360, 1624; IR (ATR) ν(cm^{-1}): 3275, 1624.

N-[3-(2-Bromophenyl)propyl]-N'-[5-methoxy-2-(1-triisopropylsilyl-1H-pyrrol-3-yl)phenyl]carbodiimide (28a)

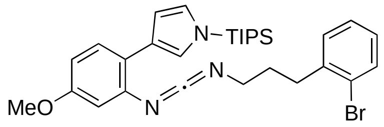

A solution of CBr_4 (1.53 g, 4.6 mmol) in CH_2Cl_2 (10 mL) was added dropwise to a solution of urea **27a** (540 mg, 0.92 mmol), PPh_3 (1.21 g, 4.6 mmol), and Et_3N (1.3 mL, 9.2 mmol) in CH_2Cl_2 (21 mL) at 0 °C. After stirring at rt for 30 min, the reaction mixture was evaporated *in vacuo*. The residue was purified by column chromatography using EtOAc/hexane 1:4 (v/v) as an eluent to give the carbodiimide **28a** (387 mg, 74%). Yellow oil. ^{1}H NMR (300 MHz, $CDCl_3$) δ 7.52 (d, J = 7.8 Hz, 1H), 7.38 (d, J = 8.5 Hz, 1H), 7.20–7.22 (m, 3H), 7.03–7.08 (m, 1H), 6.77 (dd, J = 2.8 and 1.5 Hz, 1H), 6.76 (d, J = 2.5 Hz, 1H), 6.69 (dd, J = 8.5 and 2.7 Hz, 1H), 6.63 (dd, J = 2.8 and 1.5 Hz, 1H), 3.83 (s,

3H), 3.39 (t, *J* = 7.9 Hz, 2H), 2.85 (t, *J* = 7.5 Hz, 2H), 1.94 (quin, *J* = 7.5 Hz, 2H), 1.46 (sept, *J* = 7.4 Hz, 3H), 1.12 (d, *J* = 7.4 Hz, 18H) ppm; ^{13}C NMR (75 MHz, $CDCl_3$) δ 157.9, 140.4, 137.2, 135.2, 132.8, 130.5, 129.5, 127.7, 127.4, 124.3, 123.8, 123.4, 123.1, 123.0, 110.9, 110.5, 109.9, 55.3, 45.9, 33.3, 31.2, 17.8, 11.6 ppm; HRMS (EI) *m/z*: [M^+] calcd for $C_{30}H_{40}BrN_3OSi$, 565.2124; found, 565.2128; IR (ATR) ν (cm^{-1}): 2866.

_N_-[3-(2-Iodophenyl)propyl]-_N'_-[5-methoxy-2-(1-triisopropylsilyl-1_H_-pyrrol-3-yl)phenyl]carbodiimide (28b)

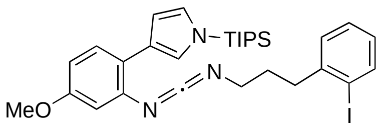

The same procedure as above was carried out using the urea **27b** (350 mg, 0.56 mmol) to give the carbodiimide **28b** (262 mg, 77%). Yellow oil; ^{1}H NMR (300 MHz, $CDCl_3$) δ 7.80 (dd, *J* = 7.8 and 1.4 Hz, 1H), 7.37 (d, *J* = 8.5 Hz, 1H), 7.25 (s, 1H), 7.20–7.22 (m, 2H), 6.88 (td, *J* = 7.8 and 1.4 Hz, 1H), 6.78 (t, *J* = 8.5 Hz, 1H), 6.75 (d, *J* = 2.8 Hz, 1H), 6.69 (dd, *J* = 8.5 and 2.7 Hz, 1H), 6.62 (dd, *J* = 2.8 and 1.5 Hz, 1H), 3.80 (s, 3H), 3.40 (t, *J* = 6.6 Hz, 2H), 2.83 (t, *J* = 6.6 Hz, 1H), 1.91 (quin, *J* = 6.6 Hz, 2H) 1.46 (sept, *J* = 7.6 Hz, 3H), 1.12 (d, *J* = 7.6 Hz, 18H) ppm; ^{13}C NMR (75 MHz, $CDCl_3$) δ 158.0, 143.7, 139.6, 137.3, 135.2, 129.63, 129.58, 128.4, 127.9, 123.9, 123.5, 123.2, 123.0, 111.0, 110.5, 110.0, 100.4, 55.4, 46.0, 37.9, 31.6, 17.9, 11.7 ppm; HRMS (EI) *m/z*: [M^+] calcd for $C_{30}H_{40}IN_3OSi$, 613.1985; found, 613.1990; IR (ATR) ν (cm^{-1}): 2133.

_N_-[3-(2-Bromophenyl)propyl]-7-methoxy-3_H_-pyrrolo[2,3-_c_]quinolin-4-amine (30a)

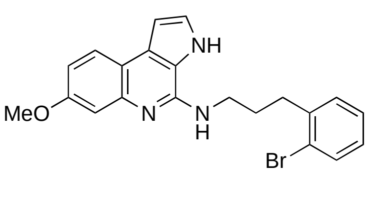

A solution of TBAF (1.0 M in THF, 0.6 mL, 0.60 mmol) was added dropwise to a solution of carbodiimide **28a** (281 mg, 0.50 mmol) in 1,2-dichlorobenzene (25 mL) under a N_2 atmosphere. After stirring at 80 °C for 30 min, the reaction mixture evaporated *in vacuo*. The residue was purified by column chromatography using EtOAc/hexane 3:2 (v/v) as an

eluent to give the pyrroloquinoline-4-amine **30a** (138 mg, 68%). Yellow oil; ^{1}H NMR (300 MHz, $CDCl_3$) δ 7.85 (d, *J* = 8.5 Hz, 1H), 7.48 (dd, *J* = 8.0 and 1.3 Hz, 1H), 7.29 (d, *J* = 2.4 Hz, 1H), 7.24 (d, *J* = 2.8 Hz, 1H), 7.14 (td, *J* = 7.6 and 1.1 Hz, 1H), 7.04–7.08 (m, 2H), 6.94 (dd, *J* = 8.5 and 2.4 Hz, 1H), 6.85 (d, *J* = 2.8 Hz, 1H), 3.85 (s, 3H), 3.62 (t, *J* = 7.2 Hz, 2H), 2.70 (t, *J* = 7.2 Hz, 1H), 1.83 (quin, *J* = 7.2 Hz, 2H) ppm; ^{13}C NMR (75 MHz, $CDCl_3$) δ 158.5, 147.6, 143.4, 140.4, 132.5, 130.0, 129.0, 127.4, 127.2, 125.4, 124.3, 123.9, 118.6, 114.9, 112.2, 106.3, 101.9, 55.3, 41.2, 33.0, 28.6 ppm; HRMS (EI) *m/z*: [M^+] calcd for $C_{21}H_{20}BrN_3O$, 409.0790; found, 409.0791; IR (ATR) ν (cm^{-1}): 3309.

N-[3-(2-Iodophenyl)propyl]-7-methoxy-3*H*-pyrrolo[2,3-*c*]quinolin-4-amine (30b)

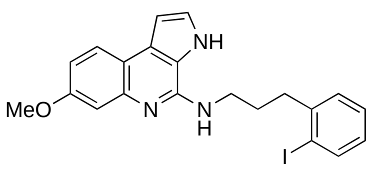

The same procedure as above was carried out using the carbodiimide **28b** (40 mg, 0.065 mmol) to give the pyrroloquinoline-4-amine **30b** (24 mg, 82%). Yellow oil; ^{1}H NMR (300 MHz, $CDCl_3$) δ 7.82 (d, *J* = 8.7 Hz, 1H), 7.67 (d, *J* = 7.6 Hz, 1H), 7.33 (d, *J* = 2.4 Hz, 1H), 7.29 (d, *J* = 2.8 Hz, 1H), 7.09 (t, *J* = 7.6 Hz, 1H), 6.91 (dd, *J* = 8.7 and 2.4 Hz, 1H), 6.83 (d, *J* = 2.8 Hz, 1H), 6.79 (t, *J* = 7.6 Hz, 1H), 6.73 (d, *J* = 7.6 Hz, 1H), 5.46 (br s, 1H), 3.77 (s, 3H), 3.36 (t, *J* = 7.6 Hz, 2H), 2.32 (t, *J* = 7.6 Hz, 1H), 1.36 (quin, *J* = 7.6 Hz, 2H) ppm; ^{13}C NMR (75 MHz, $CDCl_3$) δ 158.5, 147.4, 143.83, 143.76, 139.3, 129.3, 129.0, 128.2, 127.7, 125.1, 123.8, 118.7, 115.0, 112.4, 106.6, 102.1, 100.6, 55.3, 41.2, 37.9, 29.3 ppm; HRMS (EI) *m/z*: [M^+] calcd for $C_{21}H_{20}IN_3O$, 457.0651; found, 457.0653; IR (ATR) ν (cm^{-1}): 3313.

7-Methoxy-4-(1,2,3,4-tetrahydroquinolin-1-yl)-3*H*-pyrrolo[2,3-*c*]quinoline (31)

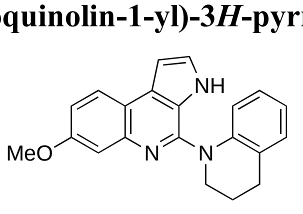

A mixture of pyrroloquinoline **30a** (147 mg, 0.36 mmol), $Pd(OAc)_2$ (4.0 mg, 0.018 mmol), $Cu(OAc)_2$ (33 mg, 0.18 mmol), and K_2CO_3 (248 mg, 1.8 mmol) in toluene (7 mL) was

refluxed for 23 h under a N_2 atmosphere. The reaction mixture was filtered through Celite pad and the organic layer was evaporated *in vacuo*. The residue was purified by column chromatography using EtOAc/hexane 1:4 (*v*/*v*) as an eluent to give the tetrahydroquinoline **31** (30 mg, 25%). Yellow solid; mp = 159–160 °C; ^{1}H NMR (300 MHz, $CDCl_3$) δ 8.00 (d, *J* = 8.8 Hz, 1H), 7.82 (br s, 1H), 7.42 (d, *J* = 2.8 Hz, 1H), 7.20 (d, *J* = 7.2 Hz, 1H), 7.13 (d, *J* = 2.8 Hz, 1H), 7.10 (t, *J* = 2.8 Hz, 1H), 7.02 (td, *J* = 7.8 and 1.9 Hz, 1H), 6.95 (td, *J* = 7.2 and 1.3 Hz, 1H), 6.87–6.88 (m, 1H), 6.74 (dd, *J* = 8.8 and 1.9 Hz, 1H), 4.17 (t, *J* = 6.2 Hz, 2H), 3.95 (s, 3H), 2.91 (t, *J* = 6.2 Hz, 2H), 2.11 (sept, *J* = 6.2 Hz, 2H) ppm; ^{13}C NMR (75 MHz, $CDCl_3$) δ 158.5, 145.4, 144.2, 141.2, 130.5, 129.2, 129.0, 126.3 124.7, 123.5, 121.6, 121.1, 118.7, 116.1, 115.3, 107.6, 101.1, 55.4, 47.3, 27.5, 24.0; HRMS (EI) *m/z*:[M^+] calcd for $C_{21}H_{19}N_3O$, 329.1528; found, 329.1533.

Synthesis of 31 from 30b

The same procedure as above was carried out using the urea **30b** (90 mg, 0.196 mmol) to give the tetrahydroquinoline **31** (47 mg, 73%).

Trigonoine B (1)[4]

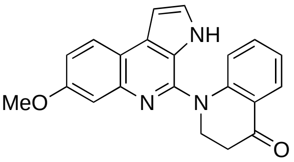

To a solution of tetrahydroquinoline **31** (27 mg, 0.082 mmol) in acetone (0.5 mL) at 0 °C was added anhydrous $MgSO_4$ (25 g, 0.21 mmol) and H_2O (0.2 mL). Subsequently, $KMnO_4$ (54 mg, 0.45 mmol) was added in small portions over 30 min. After stirring at rt for 36 h, the reaction was quenched with a saturated solution of $K_2S_2O_5$. The reaction mixture was filtered through a Celite pad, and washed with CH_2Cl_2 and H_2O. The organic layer was washed with water and brine, dried over Na_2SO_4, and evaporated *in vacuo*. The residue was purified by column chromatography using EtOAc/hexane 1:5 (v/v) to give trigonoine B (**1**) (12 mg, 43%). Yellow solid; mp = 277–278 °C; ^{1}H NMR (300 MHz, DMSO-d_6) δ 11.63 (br s, 1H), 8.20 (d, *J* = 8.8 Hz, 1H), 7.88 (dd, *J* = 7.8 and 1.7 Hz, 1H), 7.51–7.54 (m, 1H), 7.36 (d, *J* = 2.6 Hz, 1H), 7.27 (td, *J* = 7.8 and 1.7 Hz, 1H), 7.19 (dd, *J* = 8.8 and 2.6 Hz, 1H), 7.09 (dd, *J* = 2.9 and 2.0 Hz, 1H), 6.92 (t, *J* = 7.8 Hz, 1H), 6.83

(d, J = 7.8 Hz, 1H), 4.21 (t, J = 7.0 Hz, 2H), 3.88 (s, 3H), 2.89 (t, J = 7.0 Hz, 2H) ppm; ^{13}C NMR (75 MHz, DMSO-d_6) δ 193.5, 157.8, 149.2, 144.5, 143.5, 134.7, 134.7, 130.5, 128.0, 127.2, 124.1, 122.7, 121.0, 119.4, 117.1, 116.3, 116.2, 108.1, 101.0, 55.2, 48.3 ppm; HRMS (EI) *m/z*:[M$^+$] calcd for $C_{21}H_{17}N_3O_2$, 343.1321; found, 343.1327. IR (ATR) ν (cm^{-1}): 1674.

The spectral data were in agreement with the literature values.[4]

References

1. Morrison, M. D.; Hanthorn, J. J; Pratt, D. A. *Org. Lett.*, **2009**, *11*, 1051–1054.
2. Zhenhua Gu, Z.; Zakarian, A. *Org. Lett.*, **2010**, *12*, 4224–4227.
3. Nishiyama, T.; Murakami, M.; Taninaka, K.; Hamada, E.; Endo, M.; Kinou, D.; Hatae, N.; Choshi, T. *Heterocycles*, **2021**, DOI: 10.3987/COM-20-S(K)14.
4. Li, S.-F.; Di, Y.-T.; He, H.-P.; Zhang, Y.; Wang, Y.-H.; Yin, J.-L.; Tan, C.-J.; Li, S.-L.; Hao, X.-J. *Tetrahedron Lett.*, **2011**, *52*, 3186–3188.

^{1}H NMR (300 MHz, $CDCl_3$) and ^{13}C NMR (75 MHz, $CDCl_3$) spectra of **14**

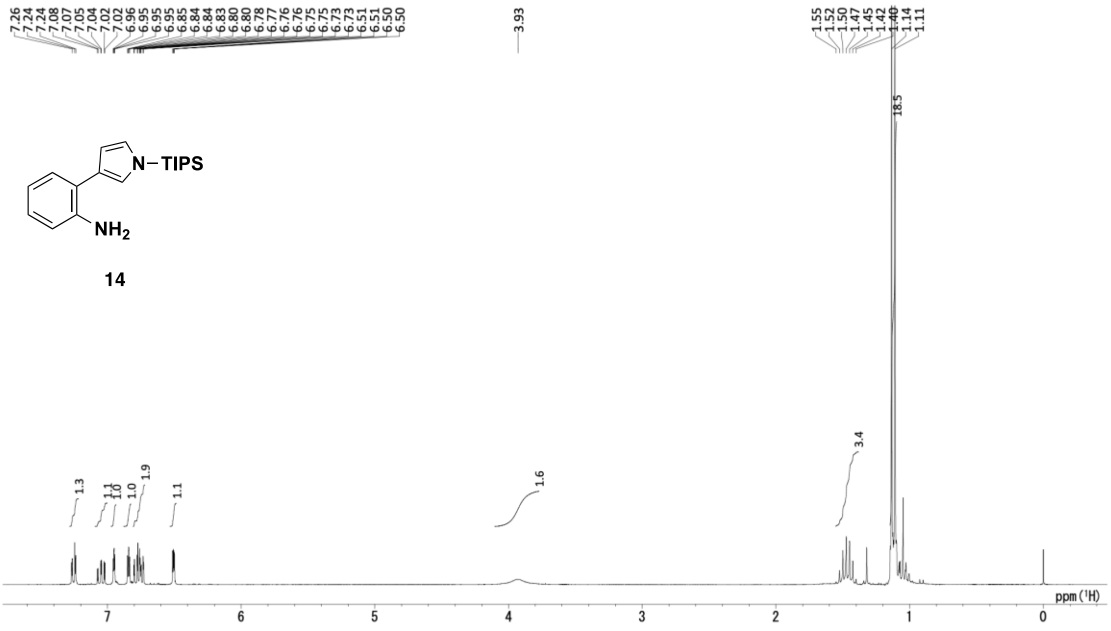

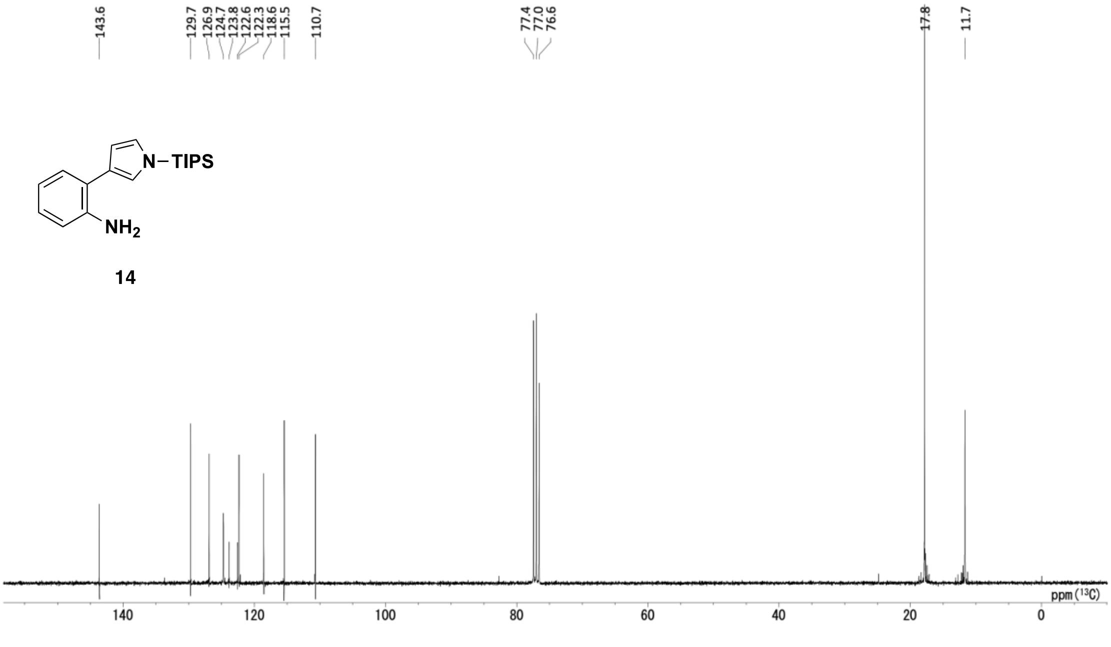

^{1}H NMR (300 MHz, $CDCl_3$) and ^{13}C NMR (75 MHz, $CDCl_3$) spectra of **15**

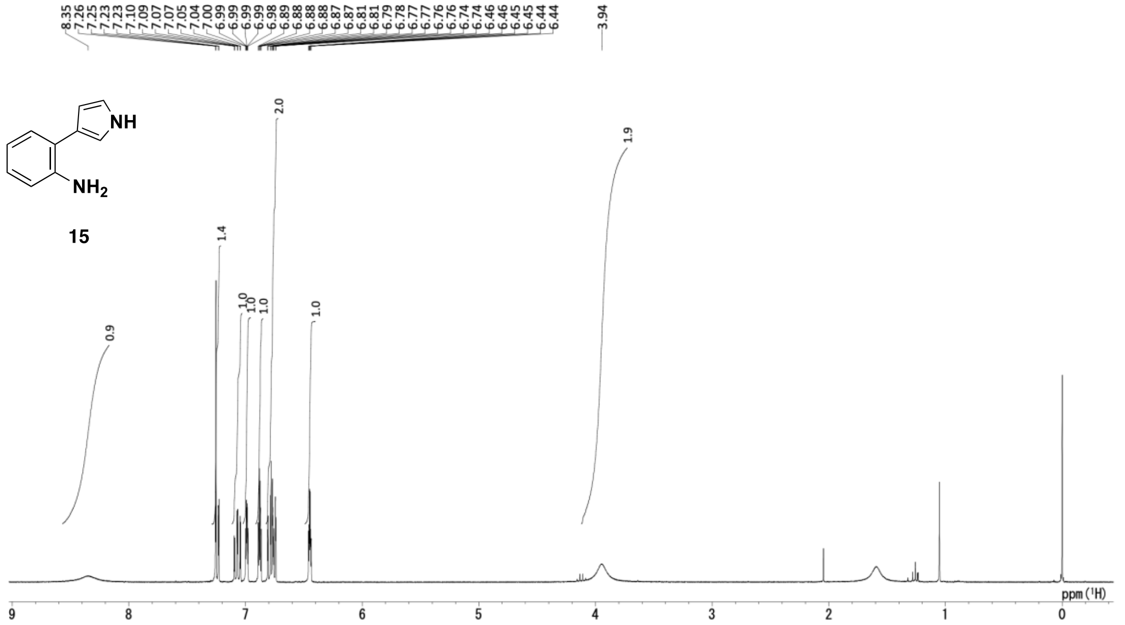

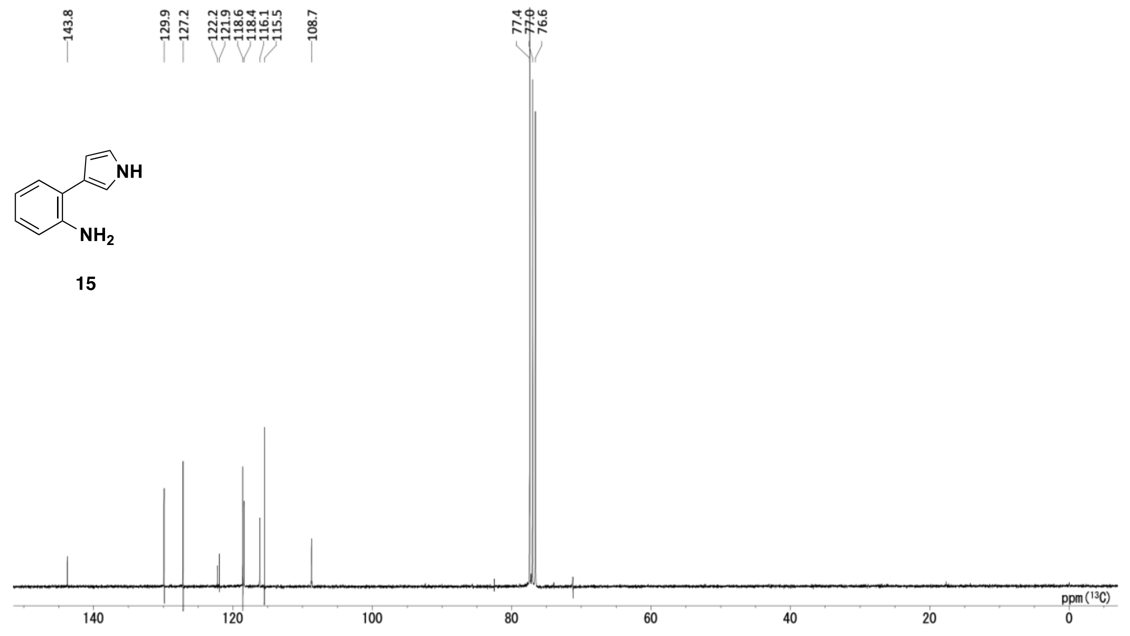

^{1}H NMR (300 MHz, DMSO-d_6) and ^{13}C NMR (75 MHz, DMSO-d_6) spectra of **16a**

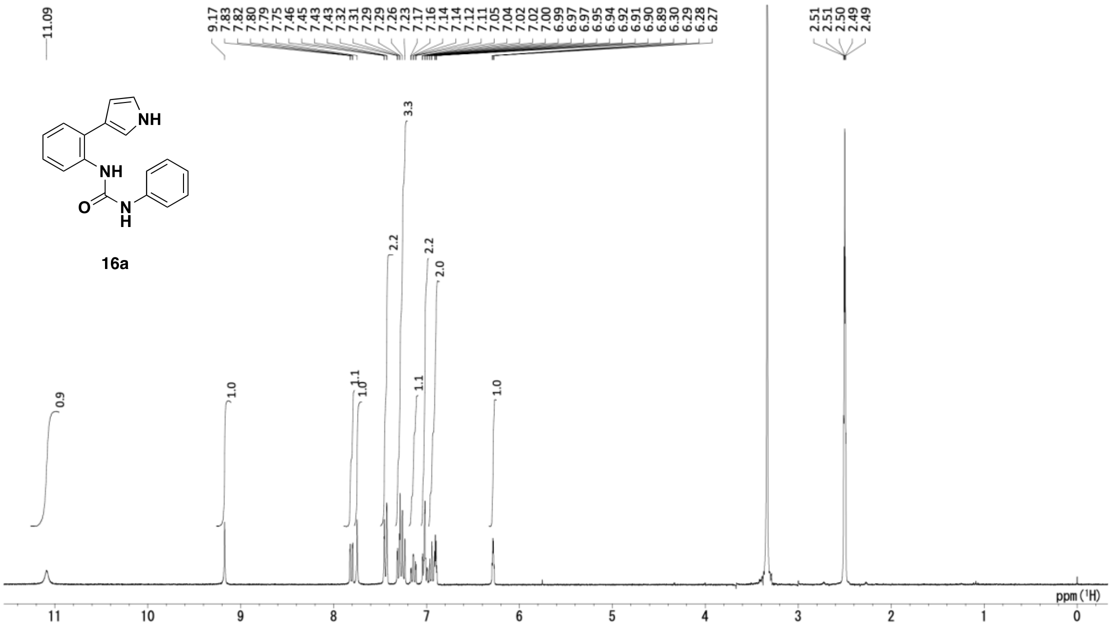

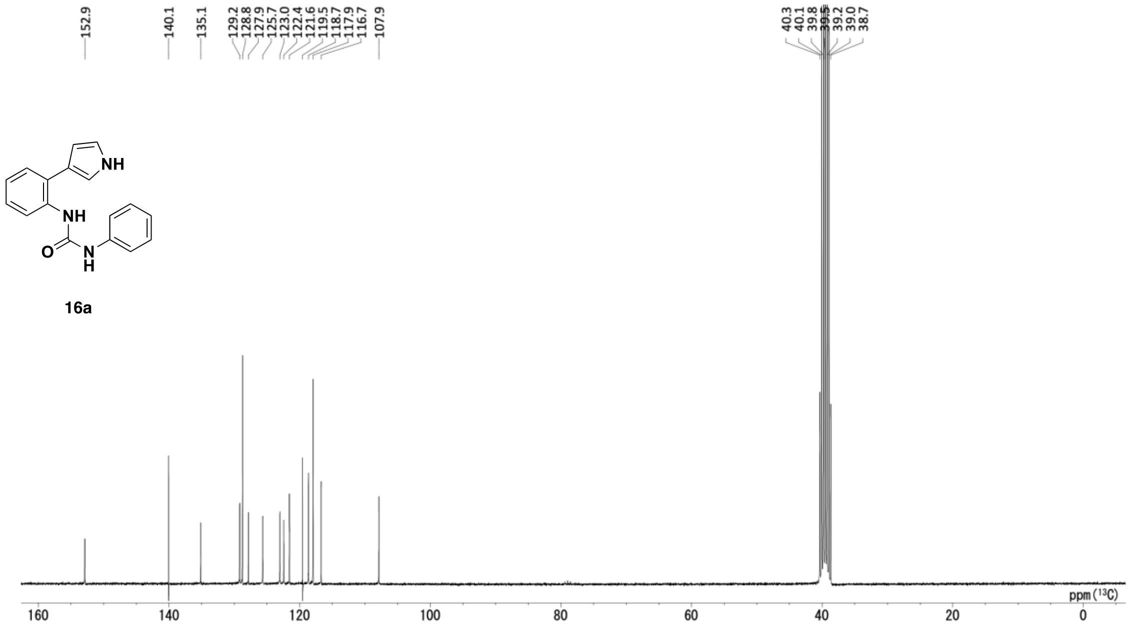

^{1}H NMR (300 MHz, DMSO-d_6) and ^{13}C NMR (75 MHz, DMSO-d_6) spectra of **16b**

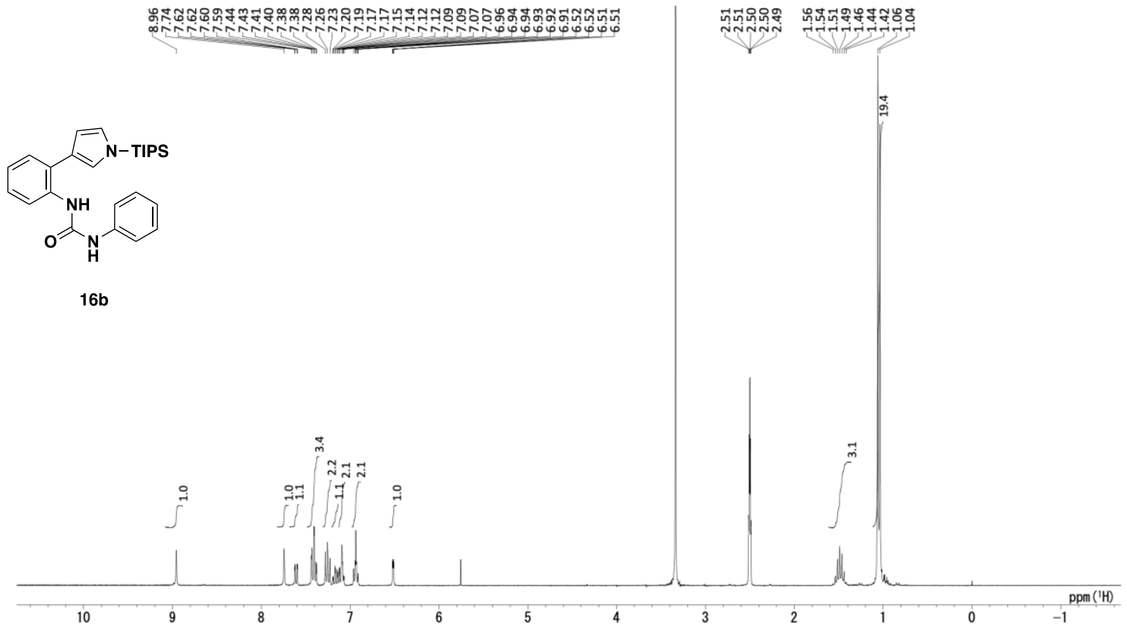

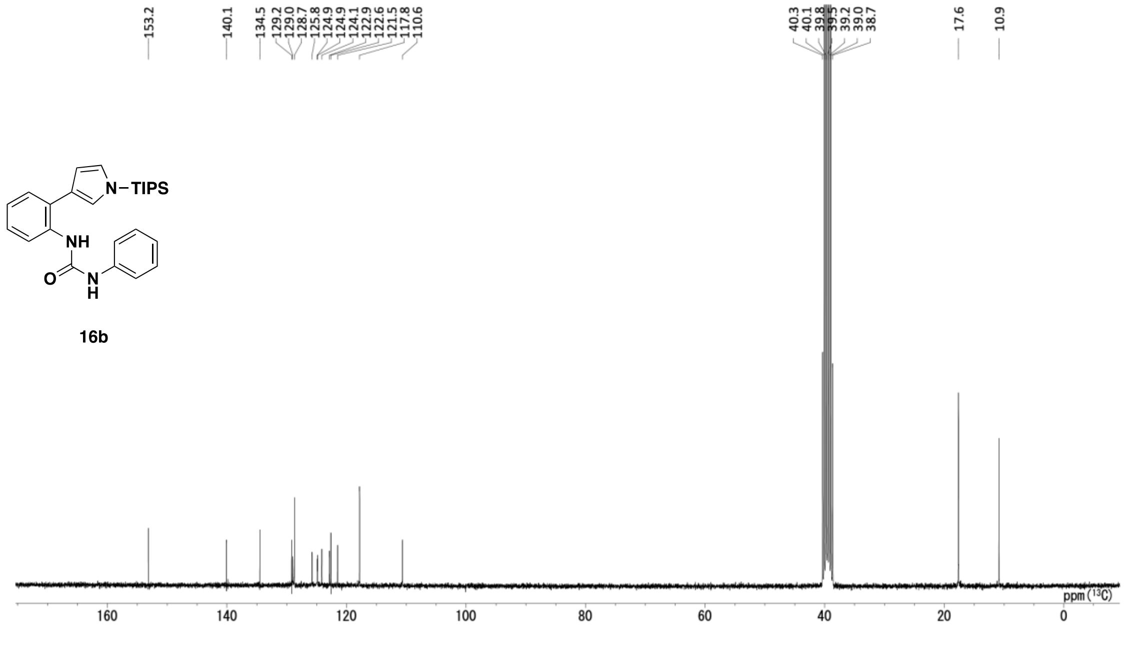

^{1}H NMR (300 MHz, $CDCl_3$) and ^{13}C NMR (75 MHz, $CDCl_3$) spectra of **17b**

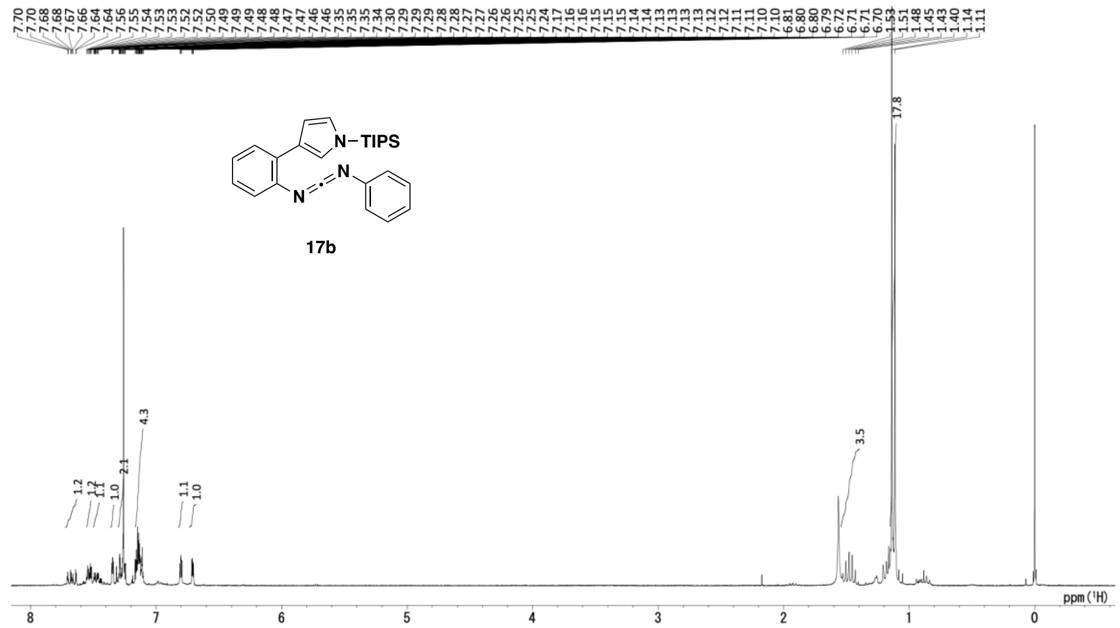

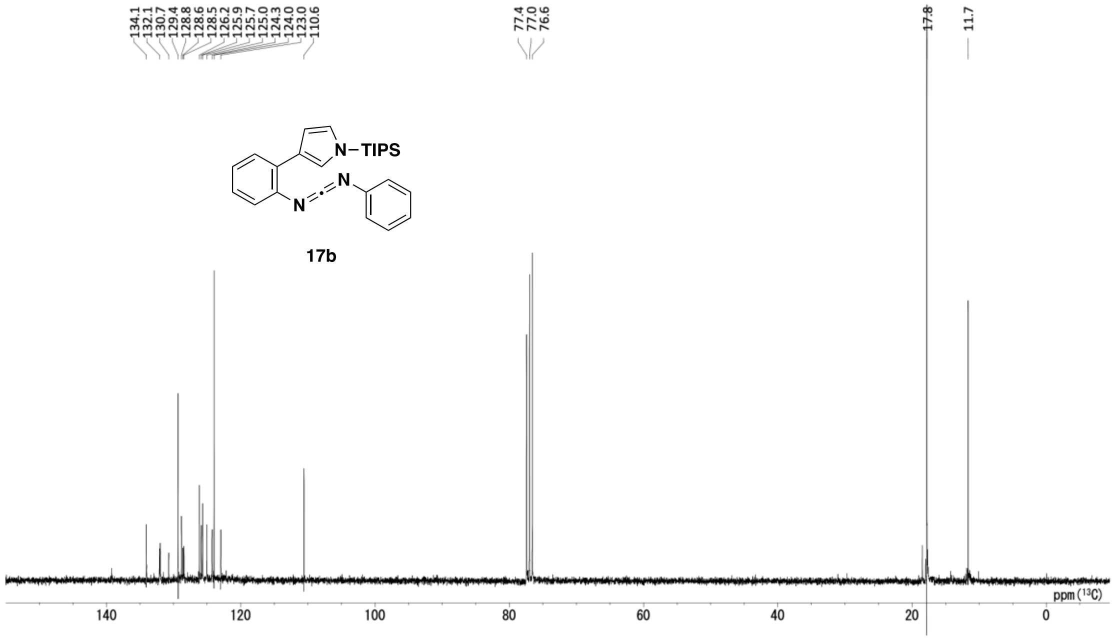

^{1}H NMR (300 MHz, $CDCl_3$) and ^{13}C NMR (75 MHz, $CDCl_3$) spectra of **18**

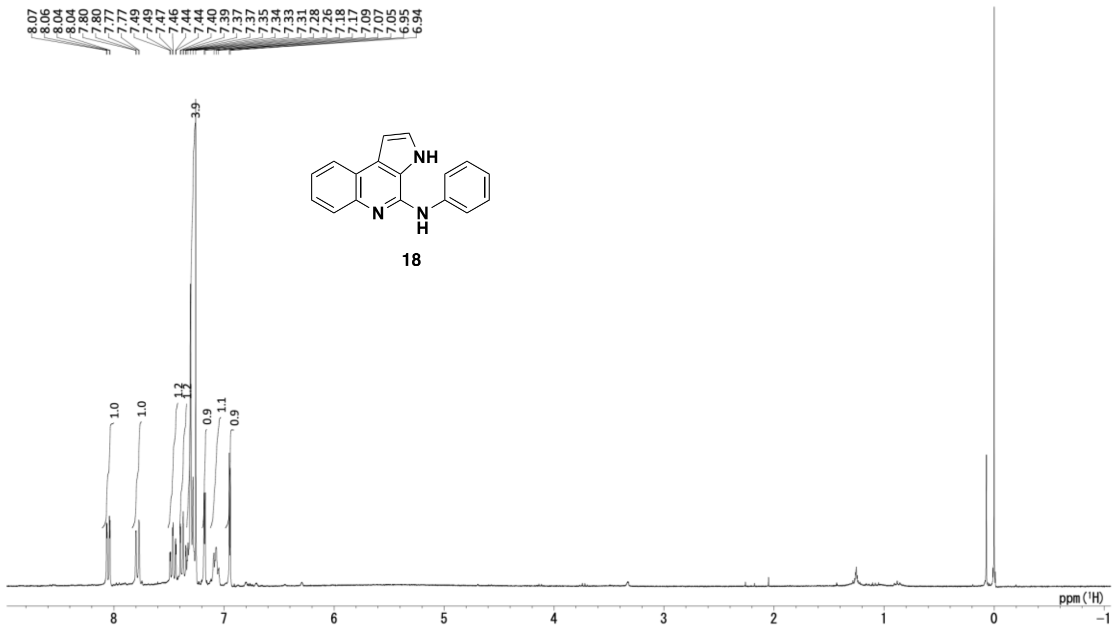

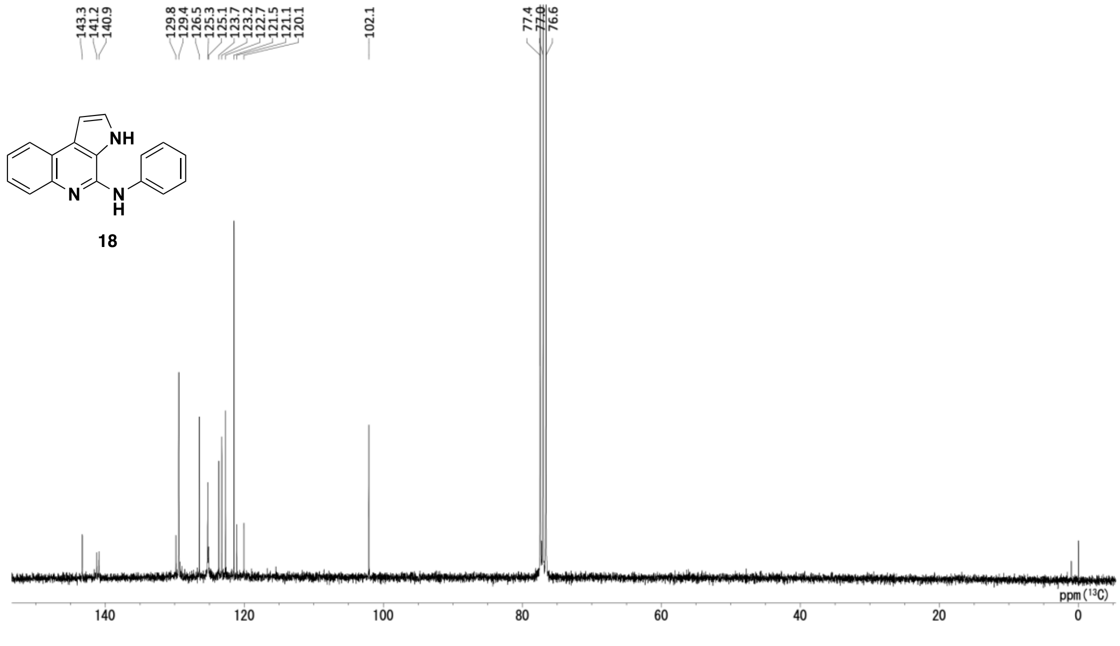

^{1}H NMR (300 MHz, $CDCl_3$) and ^{13}C NMR (75 MHz, $CDCl_3$) spectra of **19a**

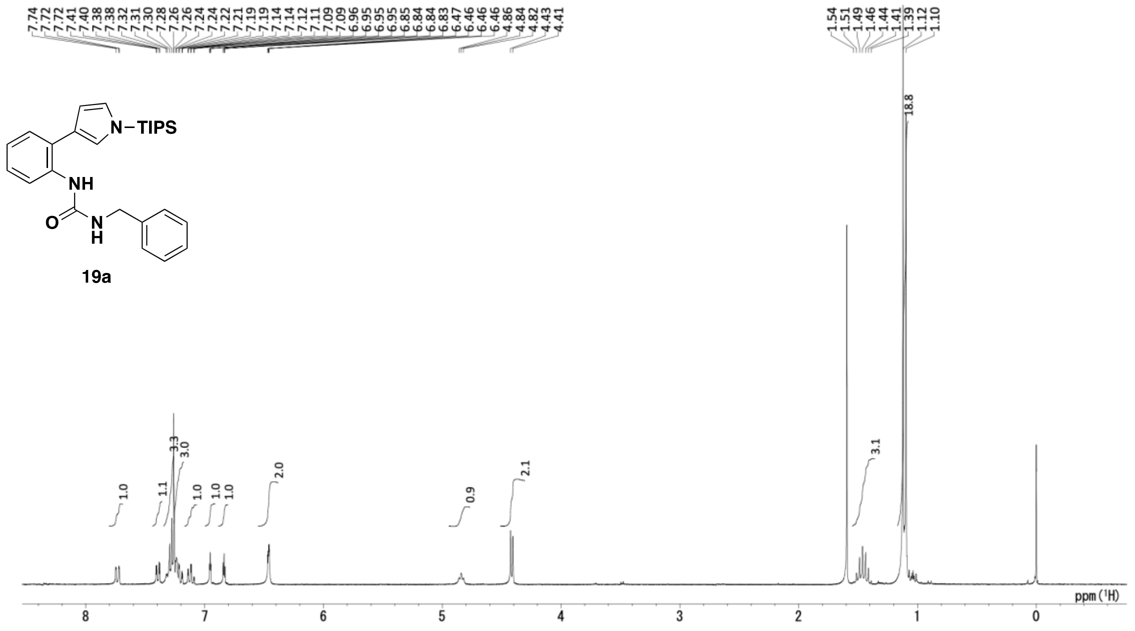

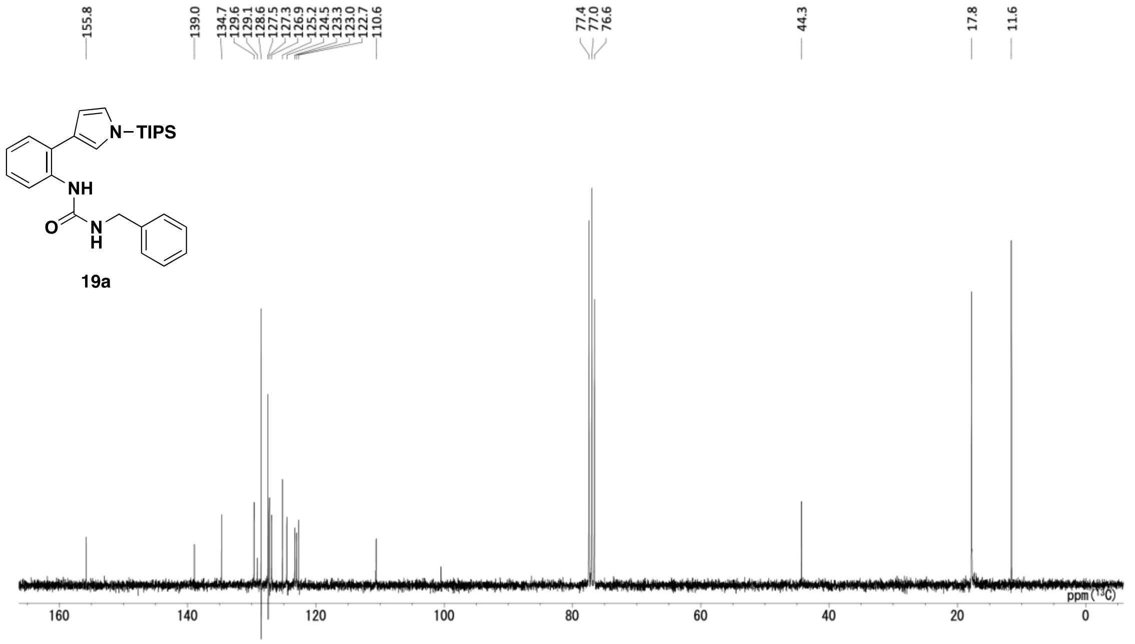

^{1}H NMR (300 MHz, $CDCl_3$) and ^{13}C NMR (75 MHz, $CDCl_3$) spectra of **19b**

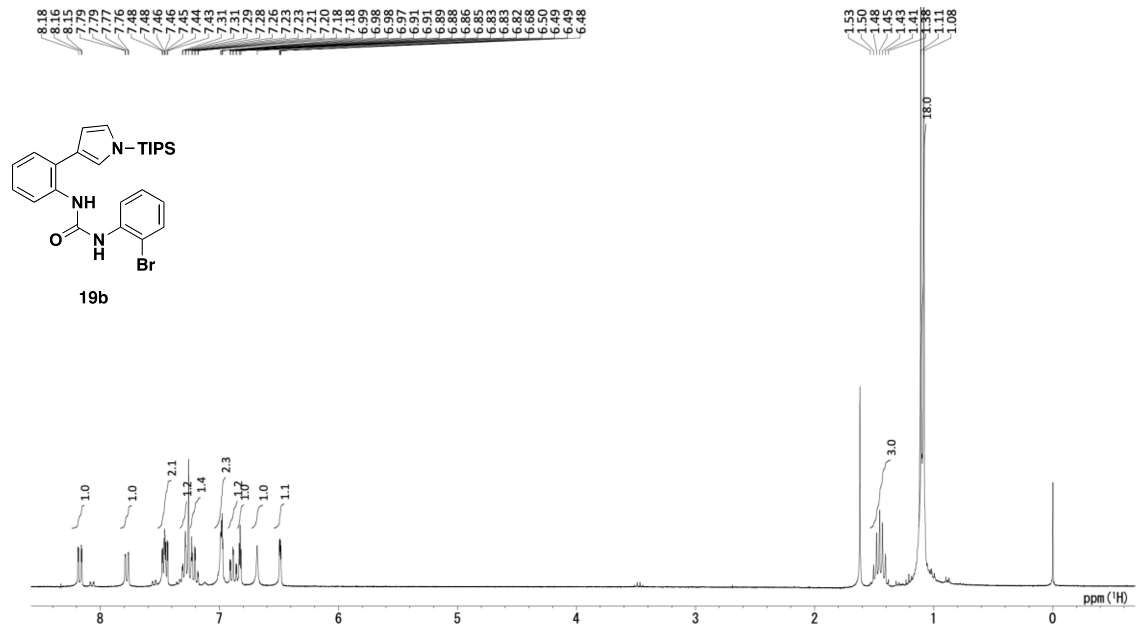

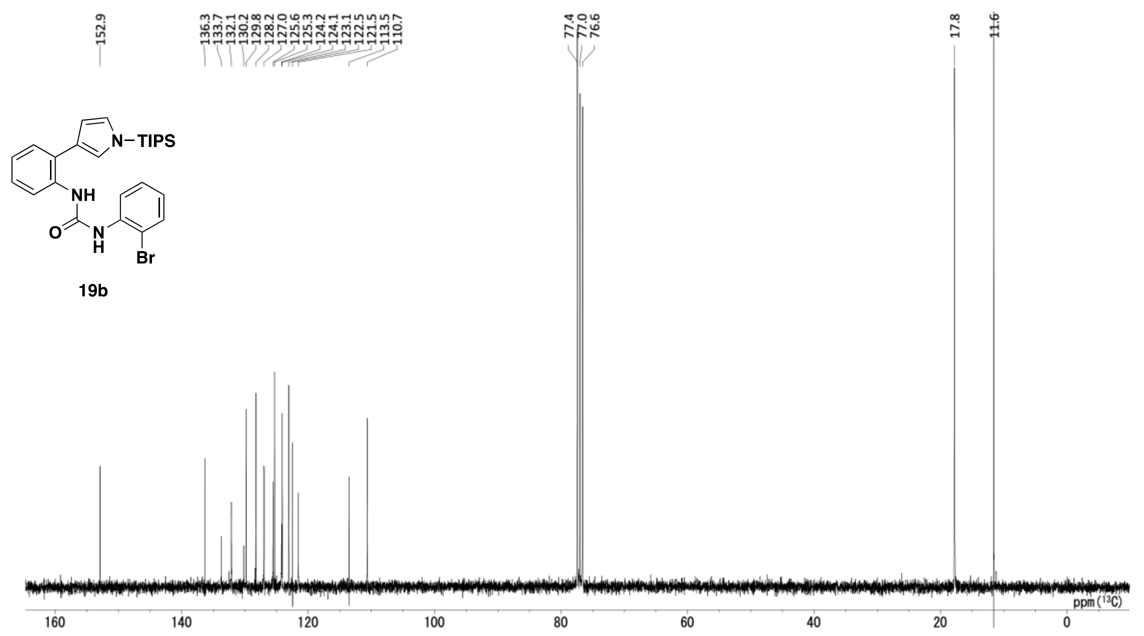

^{1}H NMR (300 MHz, $CDCl_3$) and ^{13}C NMR (75 MHz, $CDCl_3$) spectra of **19c**

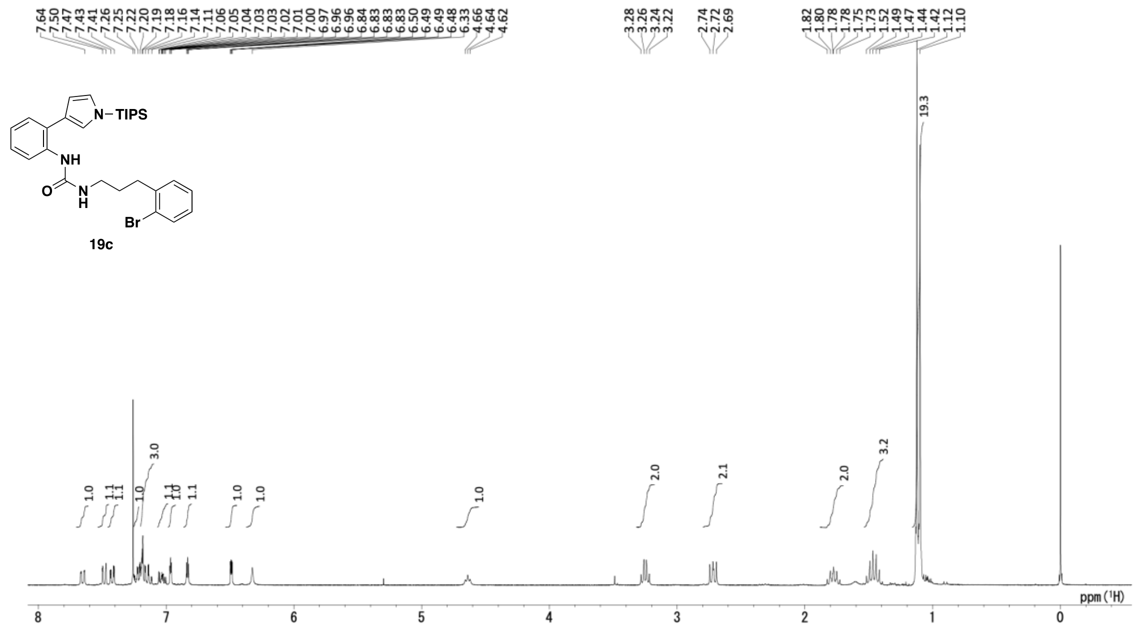

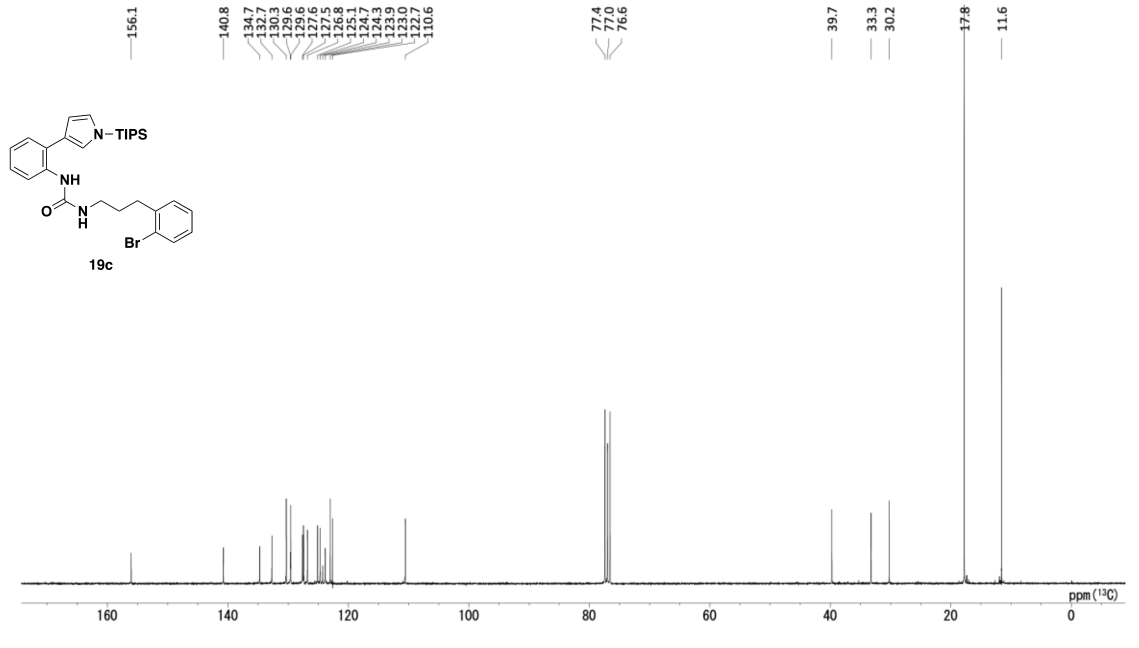

^{1}H NMR (300 MHz, $CDCl_3$) and ^{13}C NMR (75 MHz, $CDCl_3$) spectra of **19d**

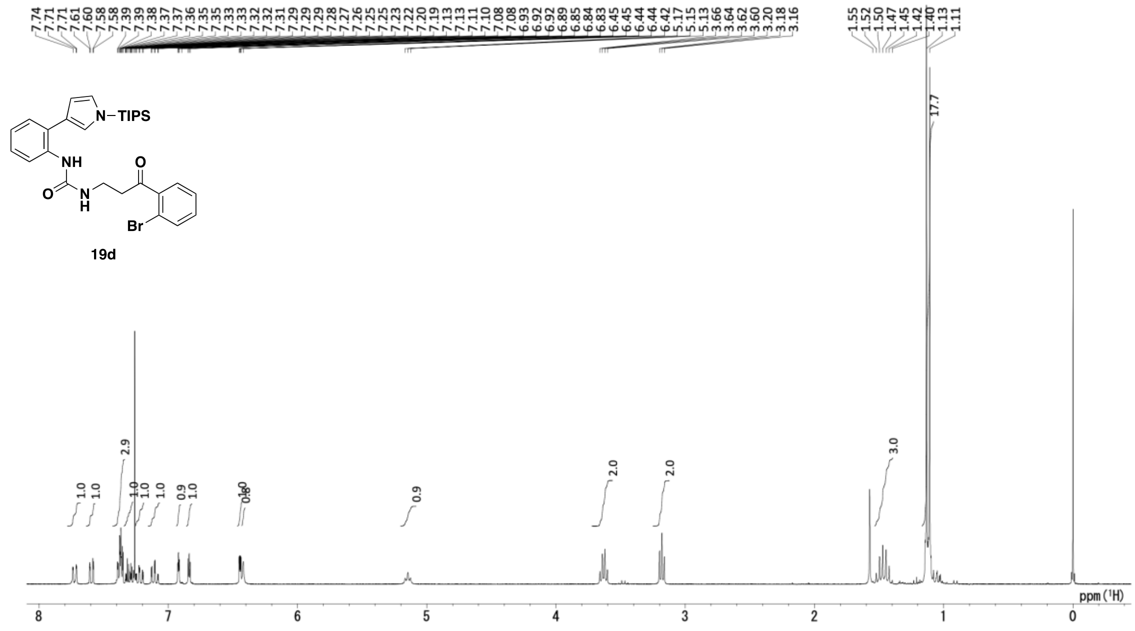

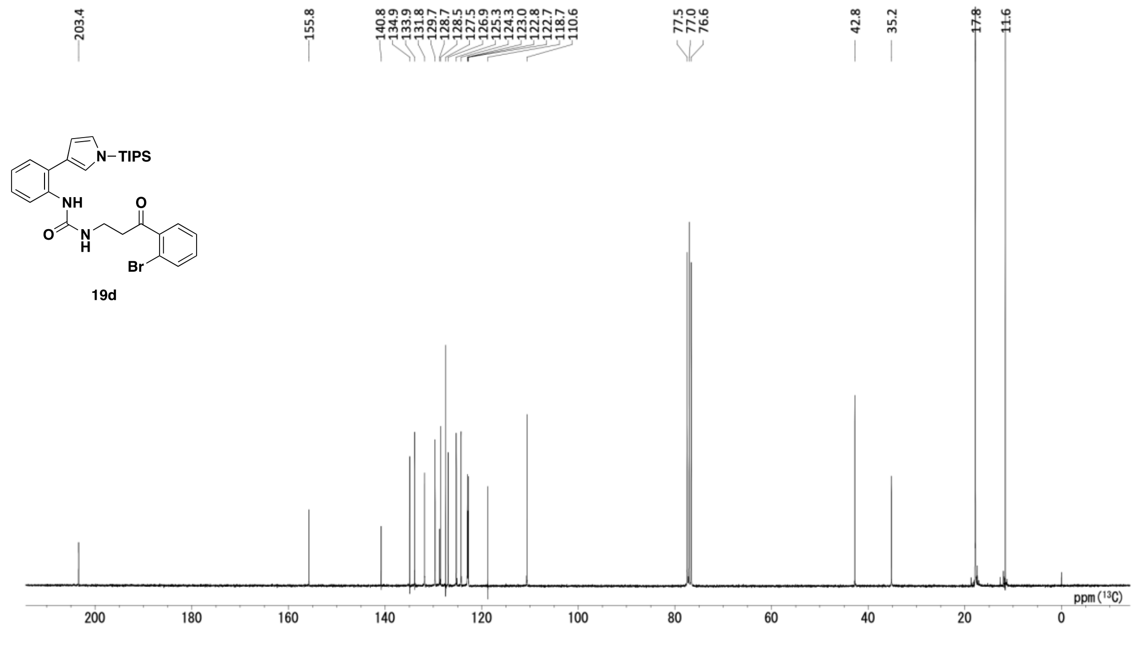

^{1}H NMR (300 MHz, $CDCl_3$) and ^{13}C NMR (75 MHz, $CDCl_3$) spectra of **20a**

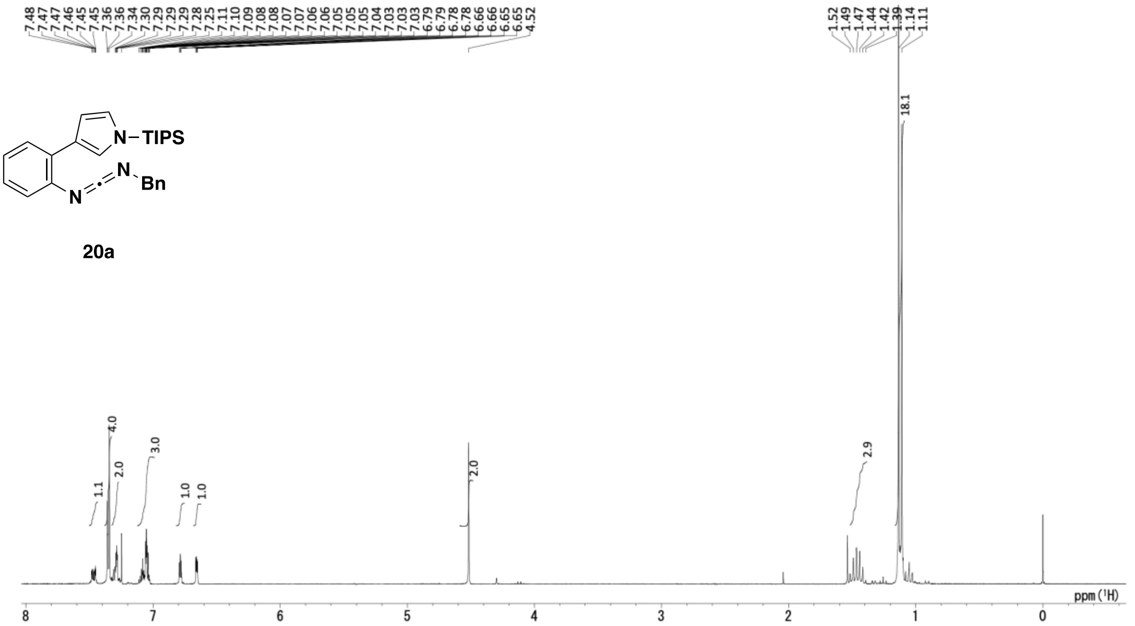

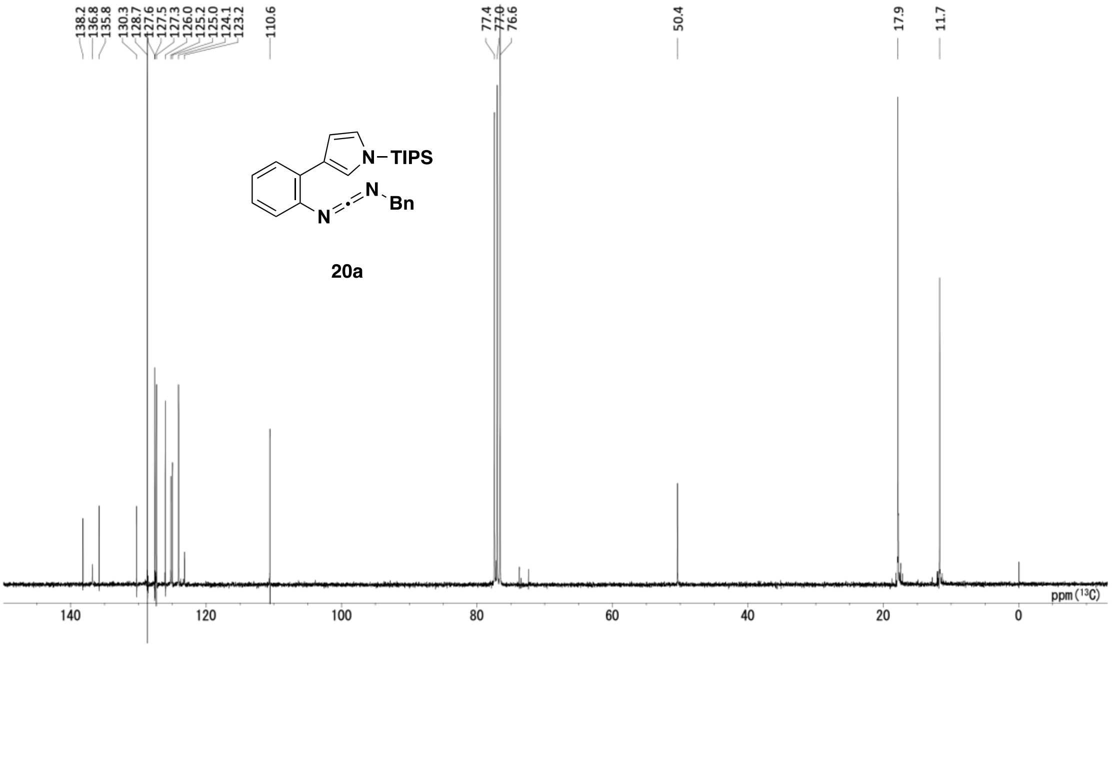

^1H NMR (300 MHz, $CDCl_3$) and ^{13}C NMR (75 MHz, $CDCl_3$) spectra of **20c**

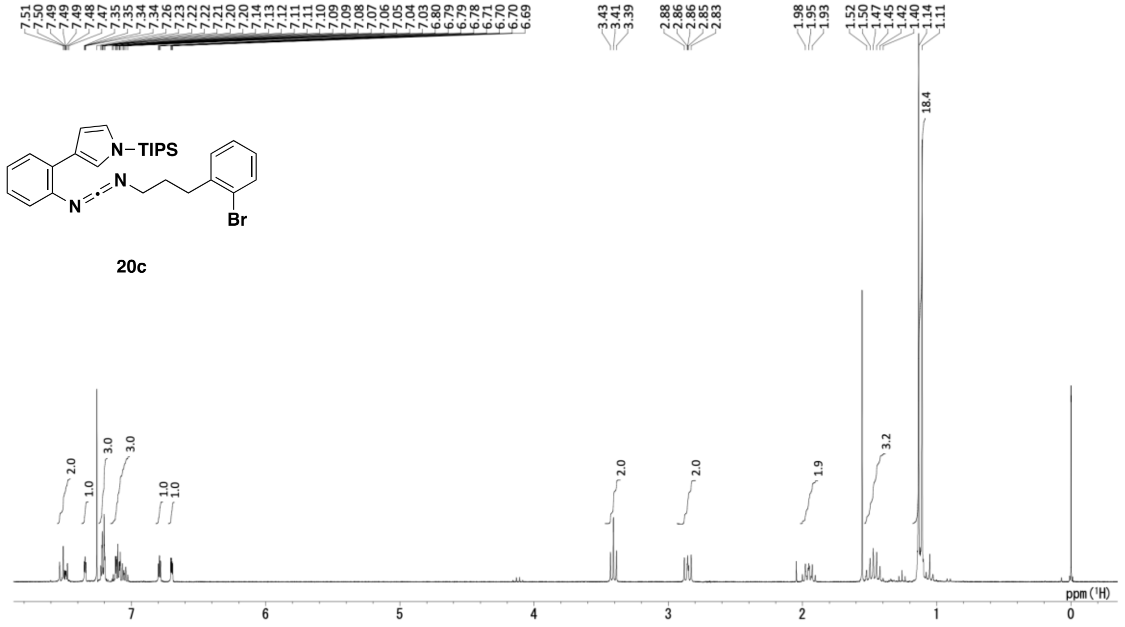

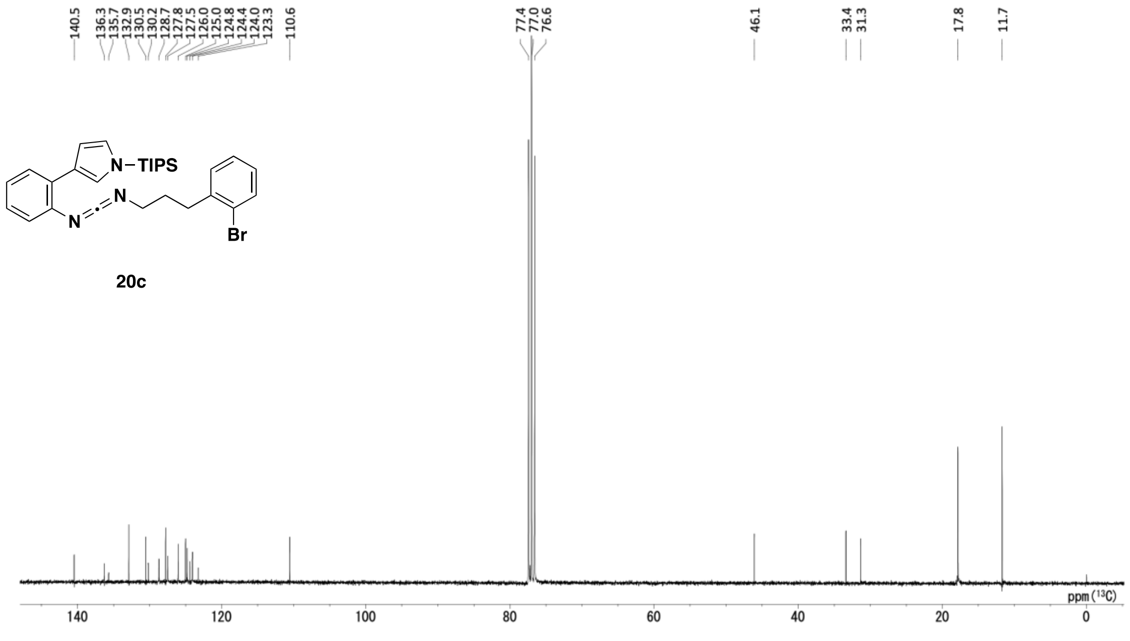

^{1}H NMR (300 MHz, $CDCl_3$) and ^{13}C NMR (75 MHz, $CDCl_3$) spectra of **20d**

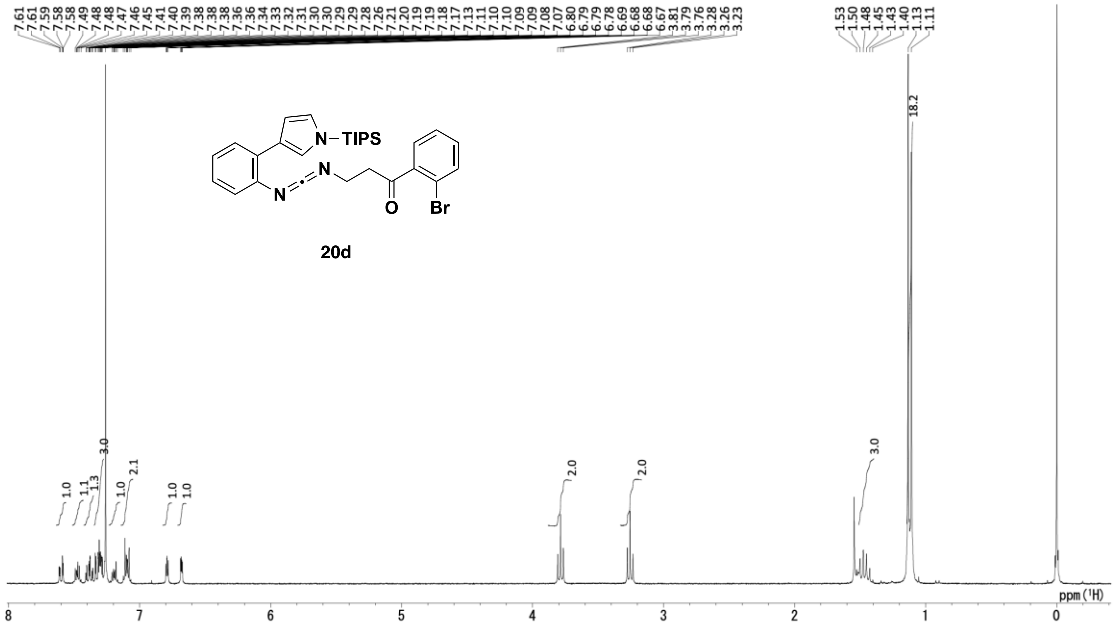

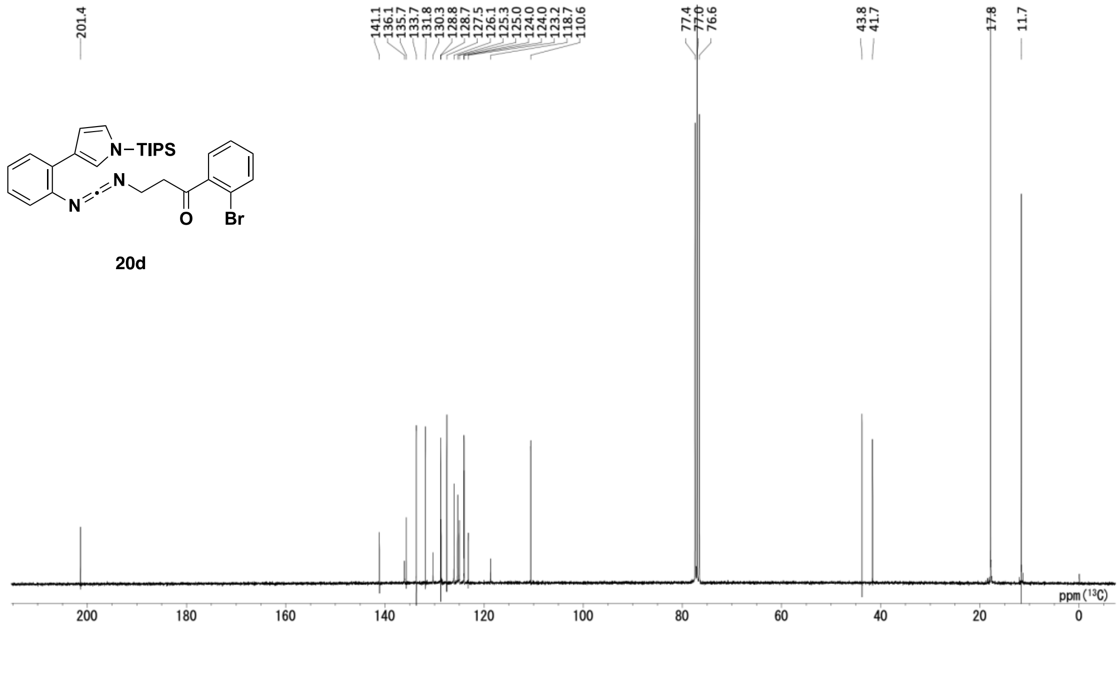

^{1}H NMR (300 MHz, $CDCl_3$) and ^{13}C NMR (75 MHz, $CDCl_3$) spectra of **22a**

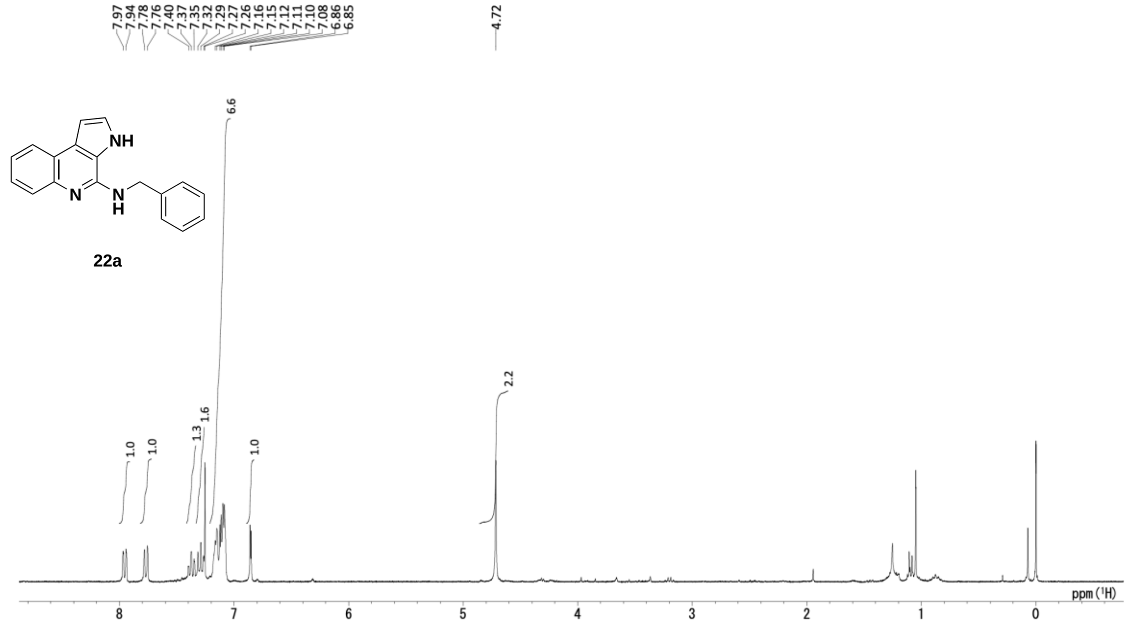

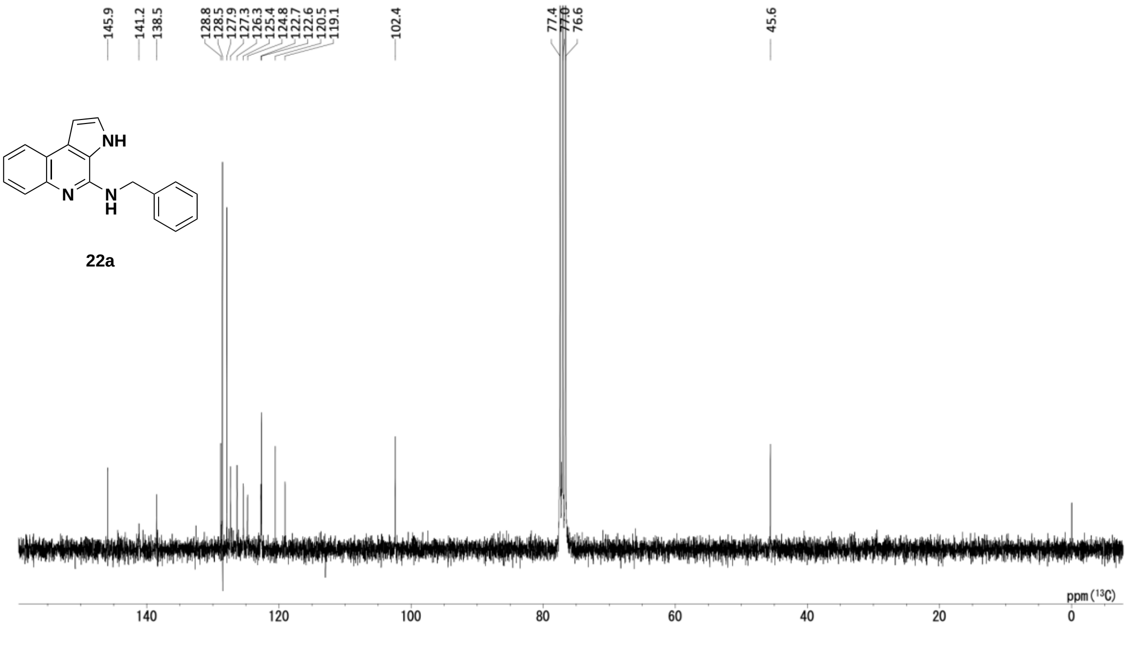

^{1}H NMR (300 MHz, DMSO-d_6) and ^{13}C NMR (75 MHz, DMSO-d_6) spectra of **22b**

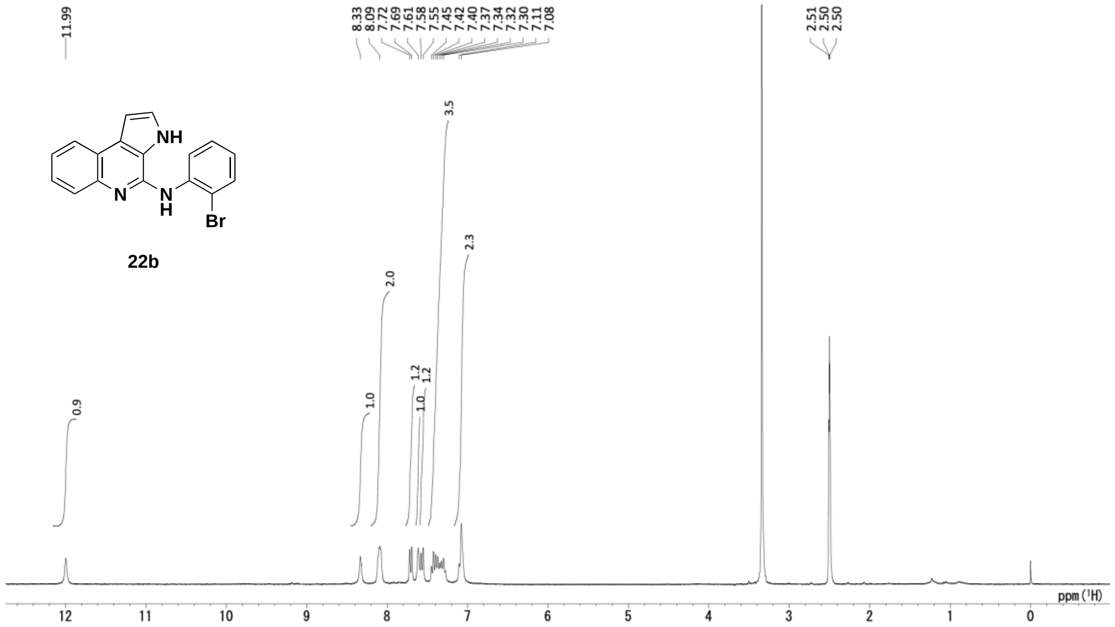

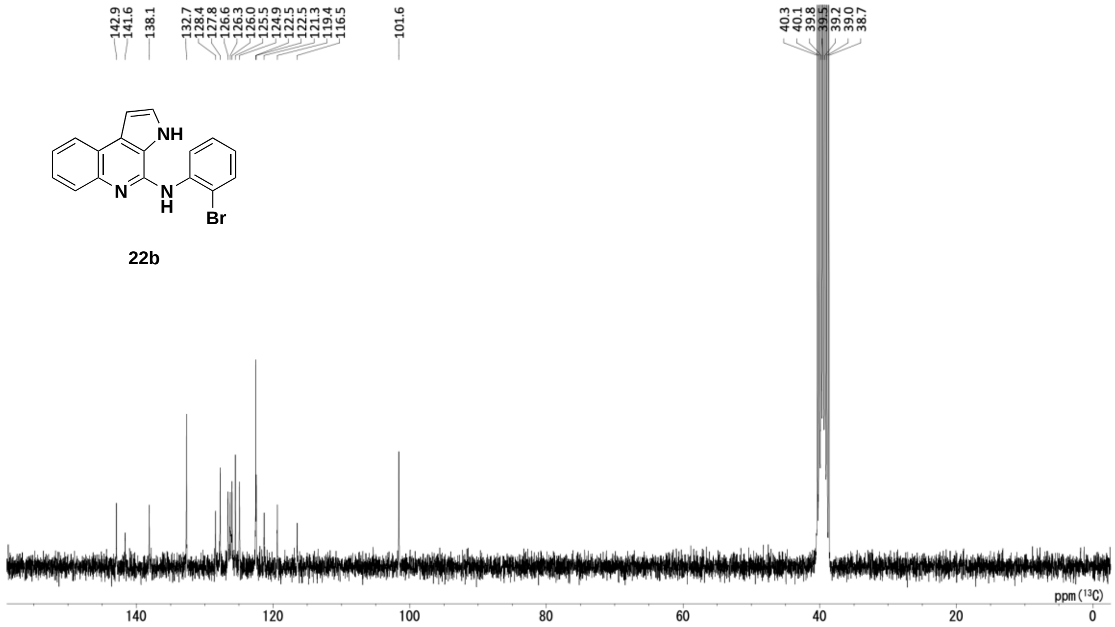

^{1}H NMR (300 MHz, $CDCl_3$) and ^{13}C NMR (75 MHz, $CDCl_3$) spectra of **22c**

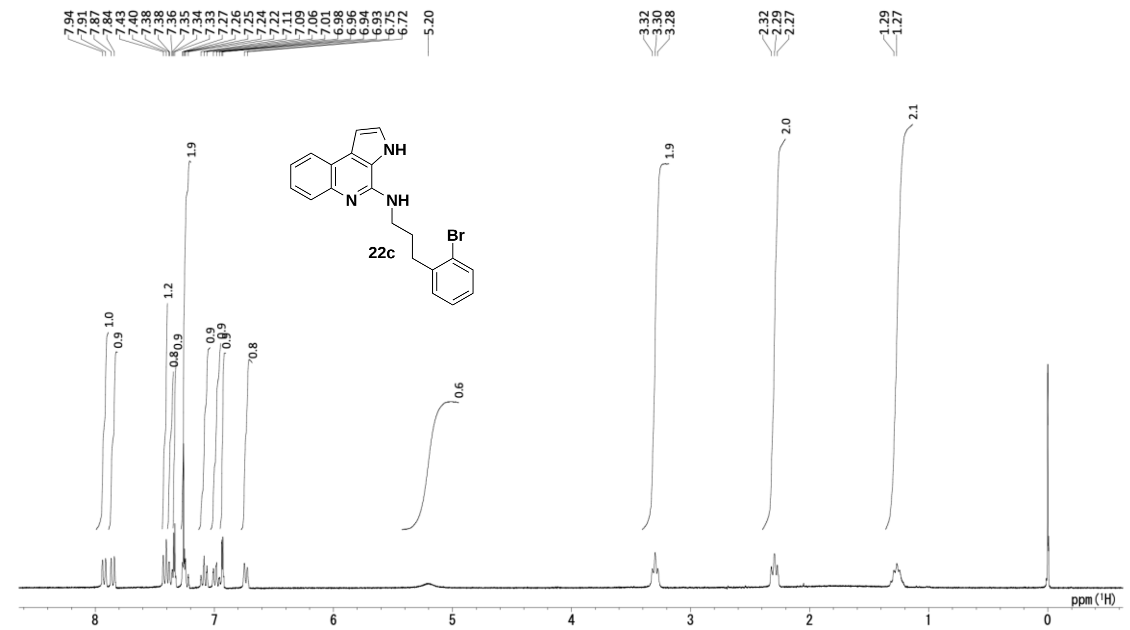

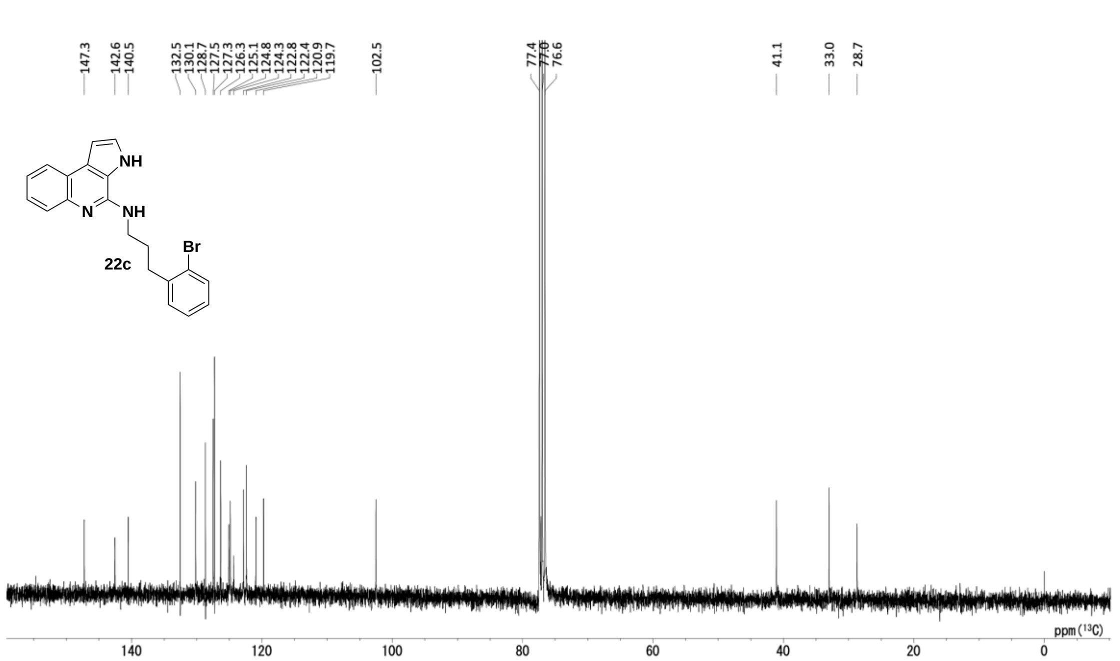

^1H NMR (300 MHz, $CDCl_3$) and ^{13}C NMR (75 MHz, $CDCl_3$) spectra of **23**

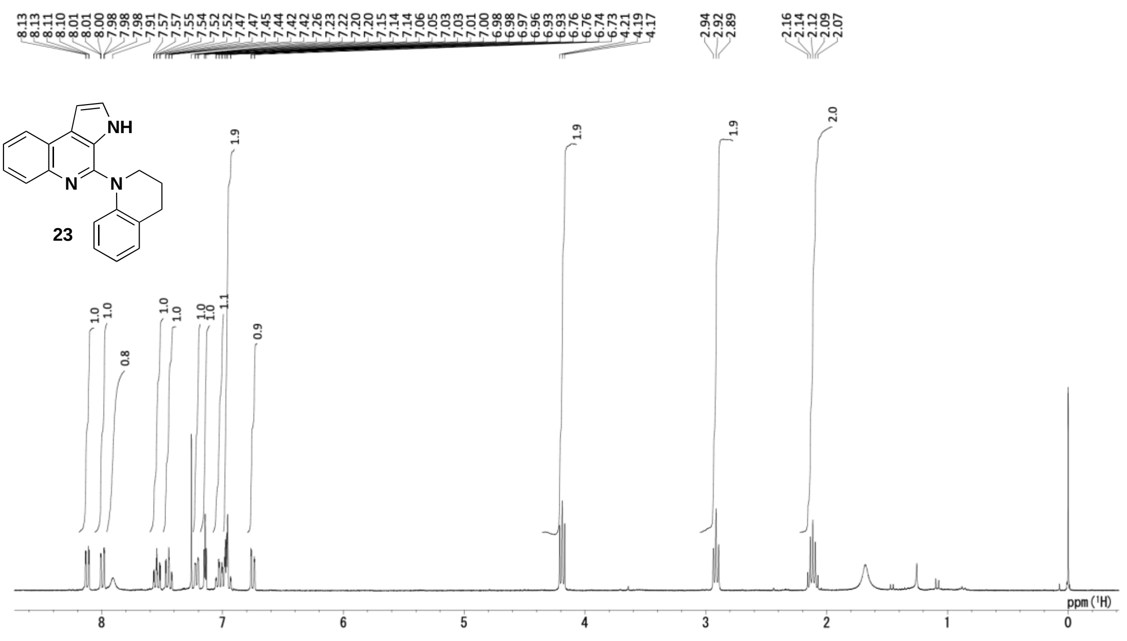

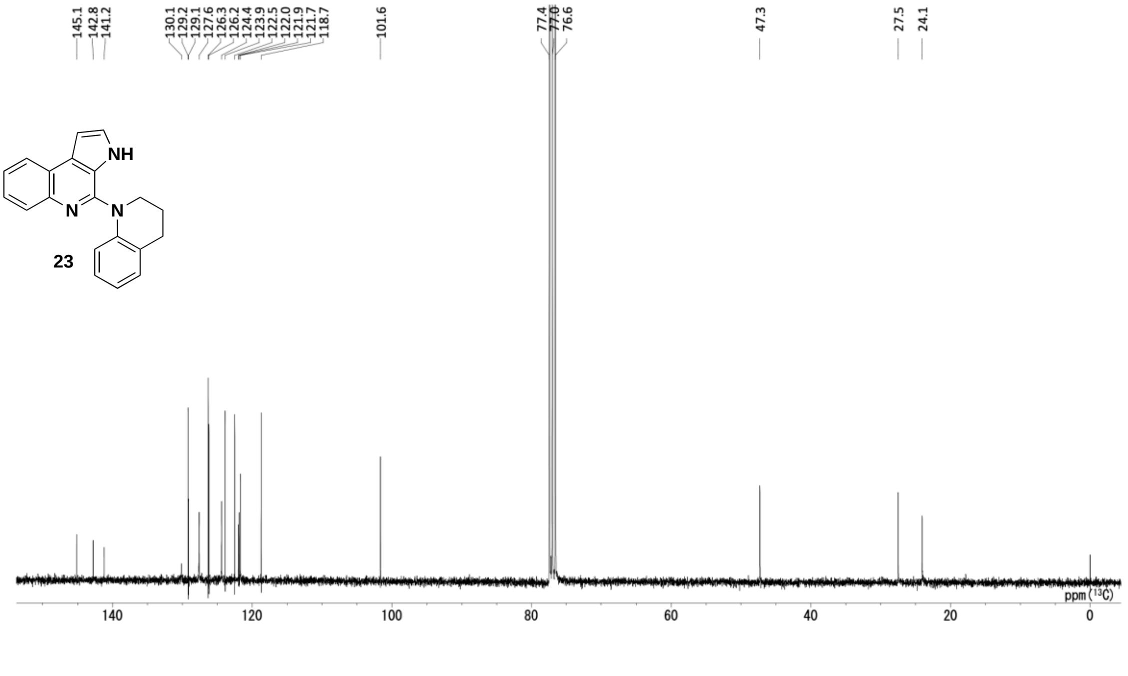

^1H NMR (300 MHz, $CDCl_3$) and ^{13}C NMR (75 MHz, $CDCl_3$) spectra of **25**

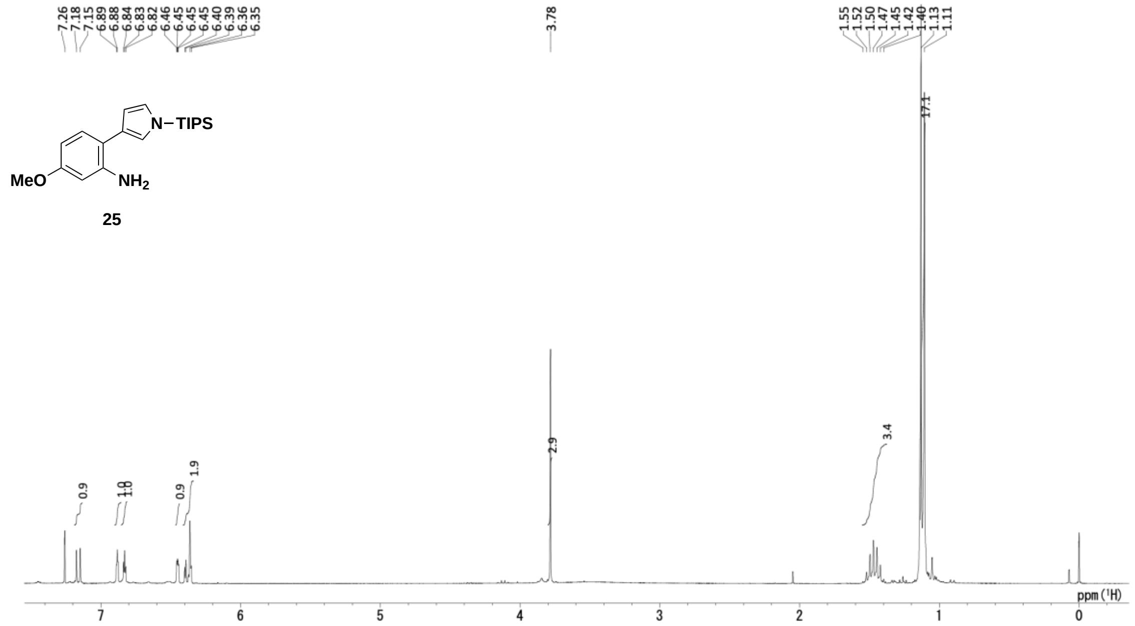

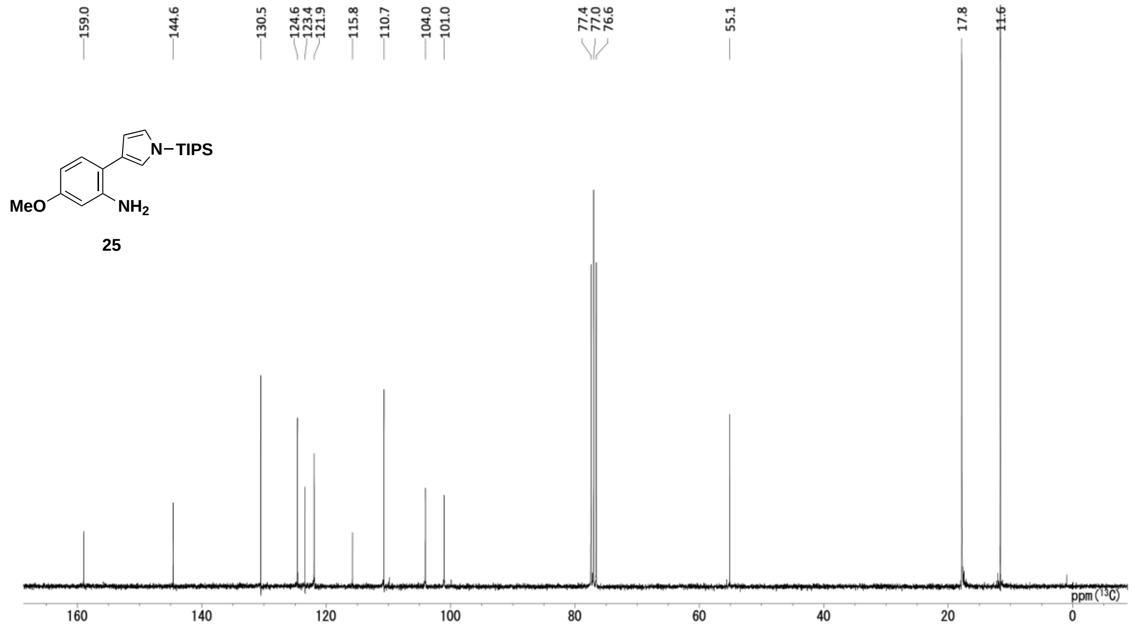

^{1}H NMR (300 MHz, $CDCl_3$) and ^{13}C NMR (75 MHz, $CDCl_3$) spectra of **27a**

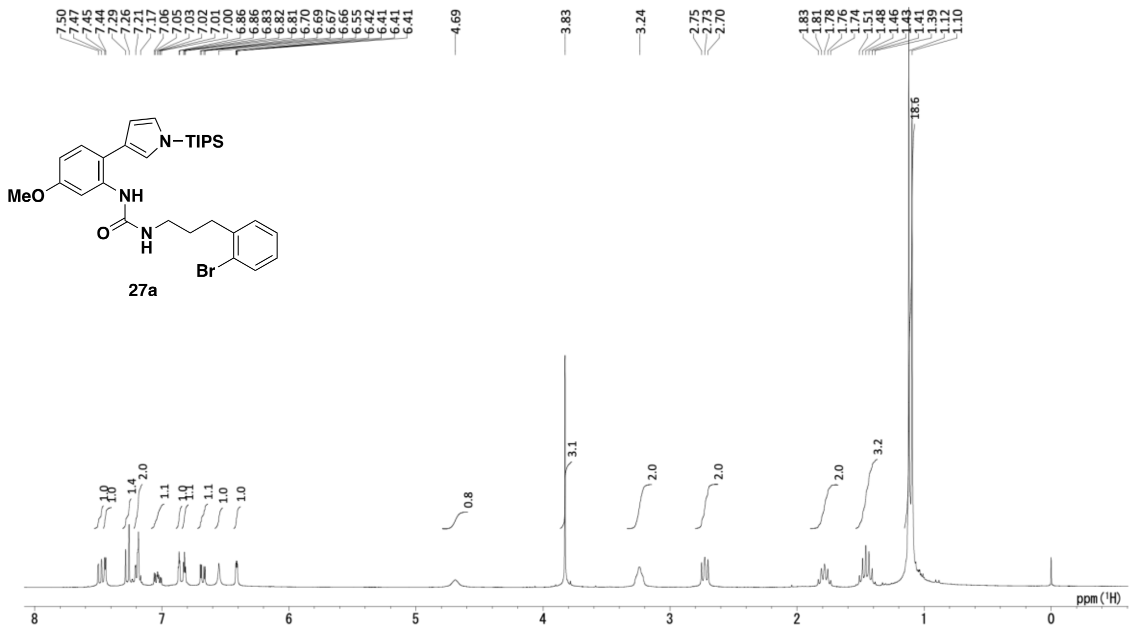

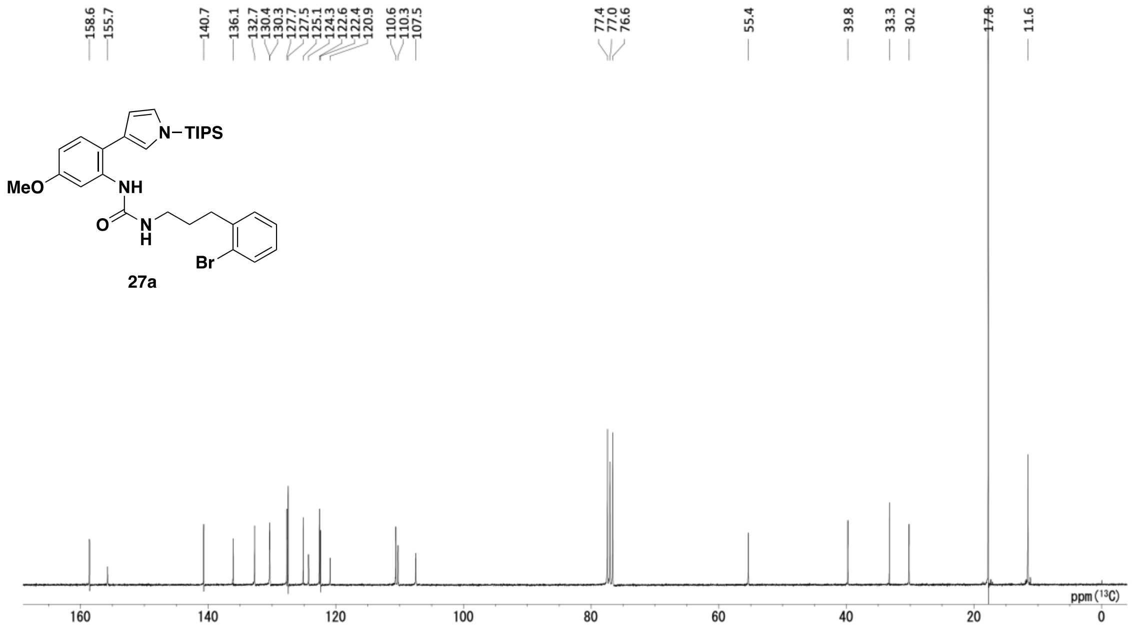

^{1}H NMR (300 MHz, $CDCl_3$) and ^{13}C NMR (75 MHz, $CDCl_3$) spectra of **27b**

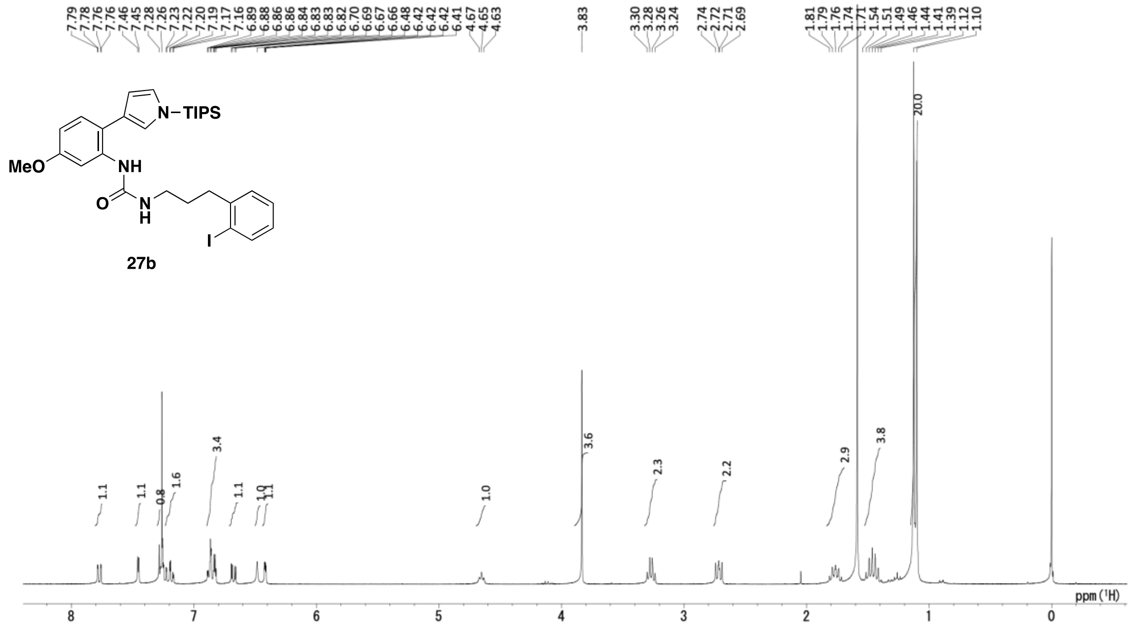

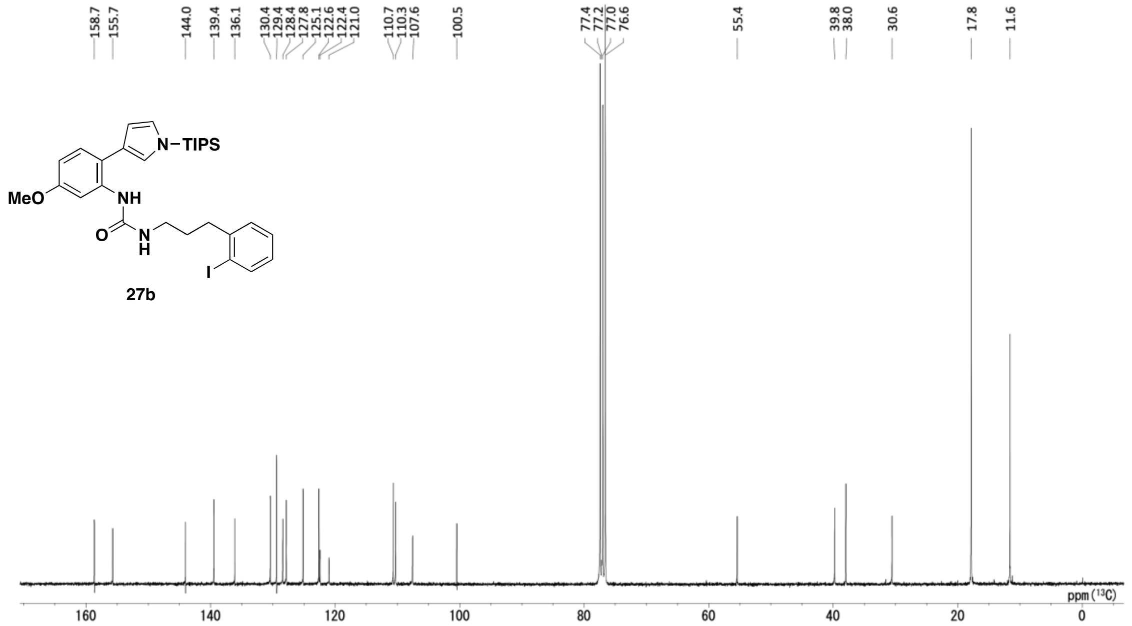

^{1}H NMR (300 MHz, $CDCl_3$) and ^{13}C NMR (75 MHz, $CDCl_3$) spectra of **28a**

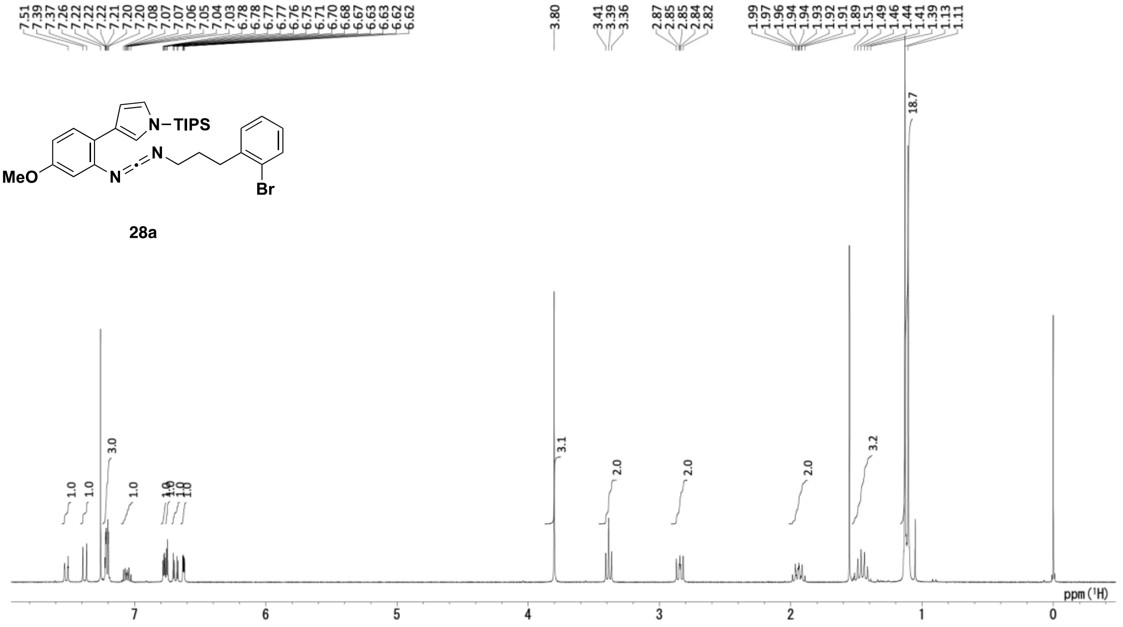

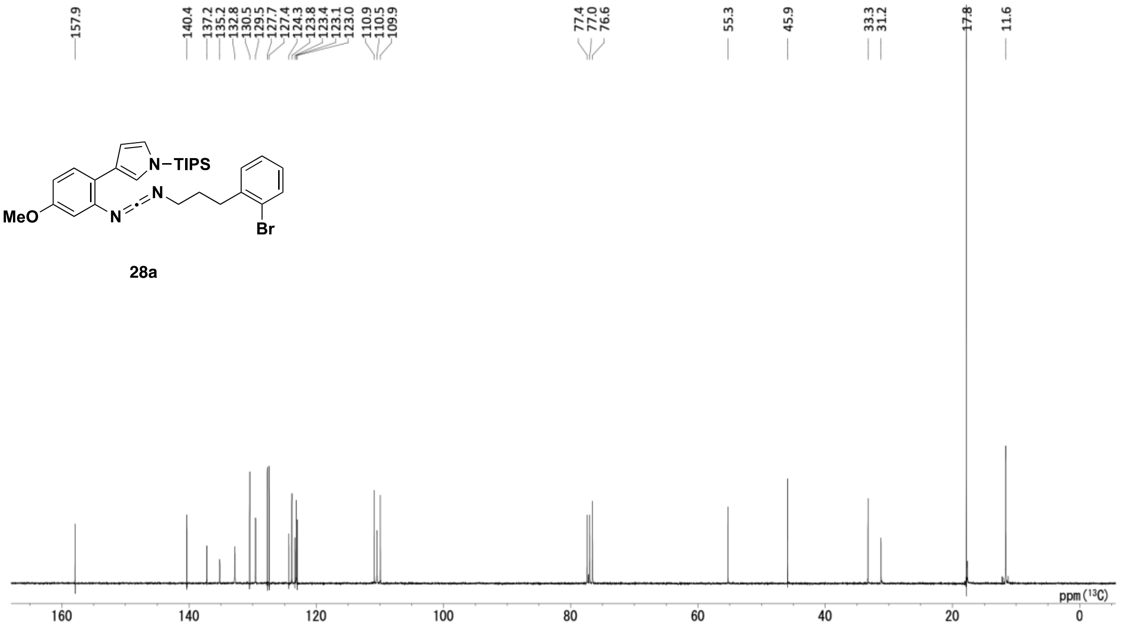

^{1}H NMR (300 MHz, $CDCl_3$) and ^{13}C NMR (75 MHz, $CDCl_3$) spectra of **28b**

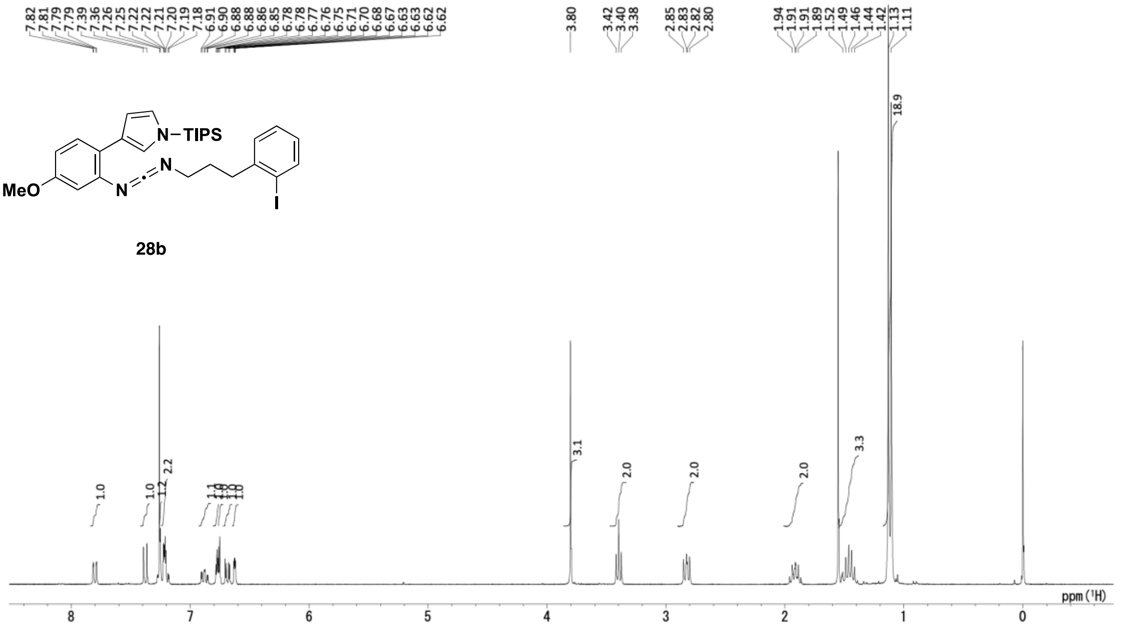

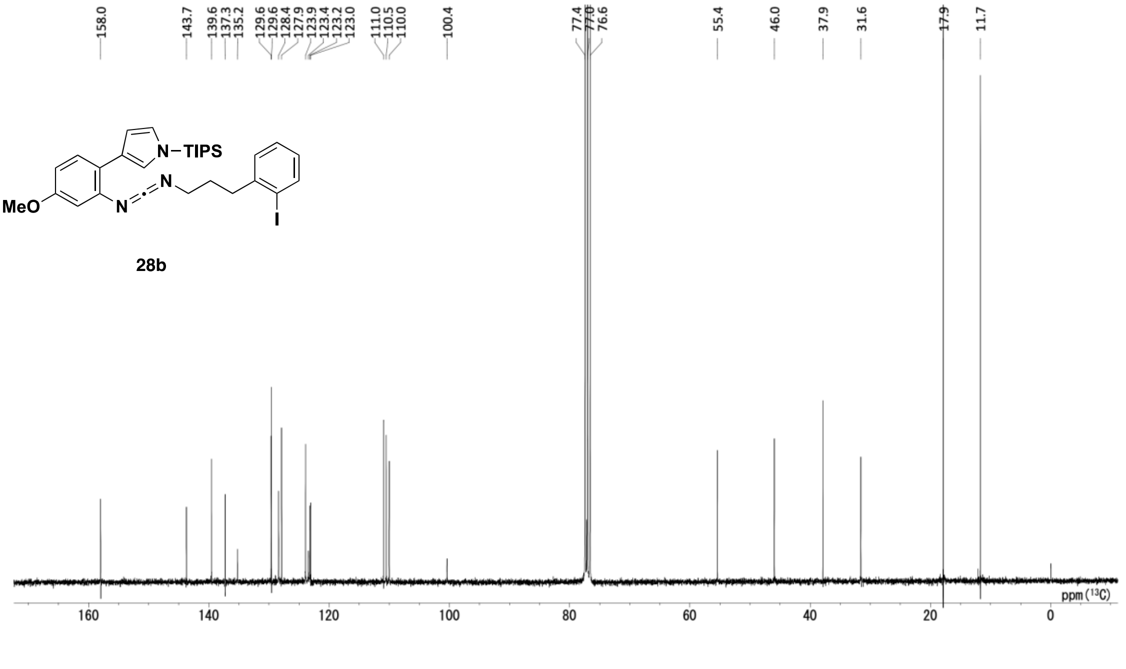

^{1}H NMR (300 MHz, $CDCl_3$) and ^{13}C NMR (75 MHz, $CDCl_3$) spectra of **30a**

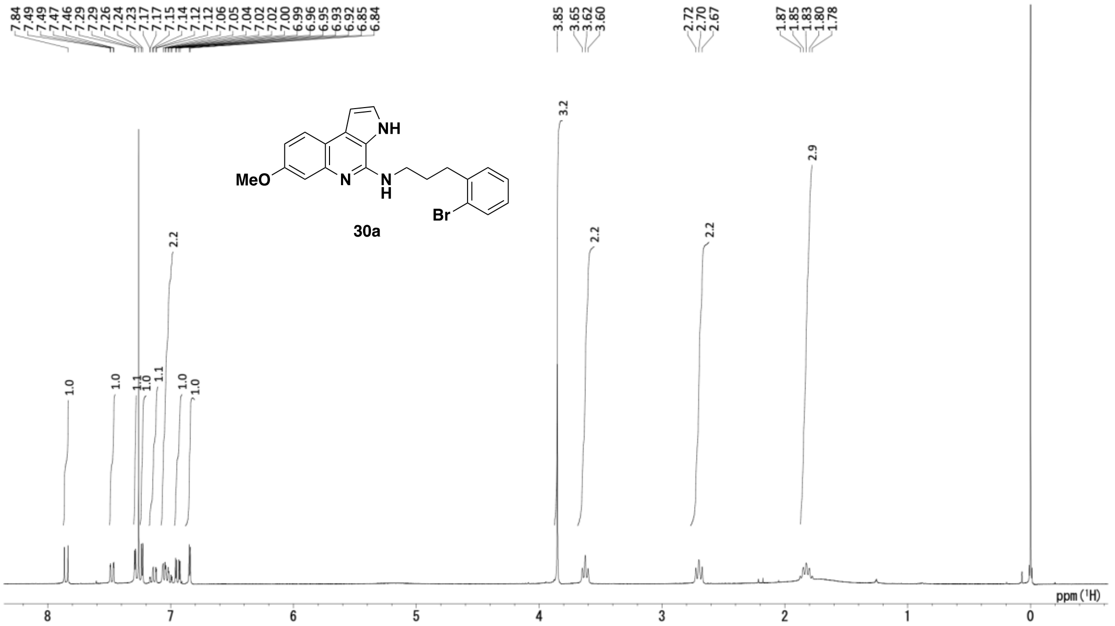

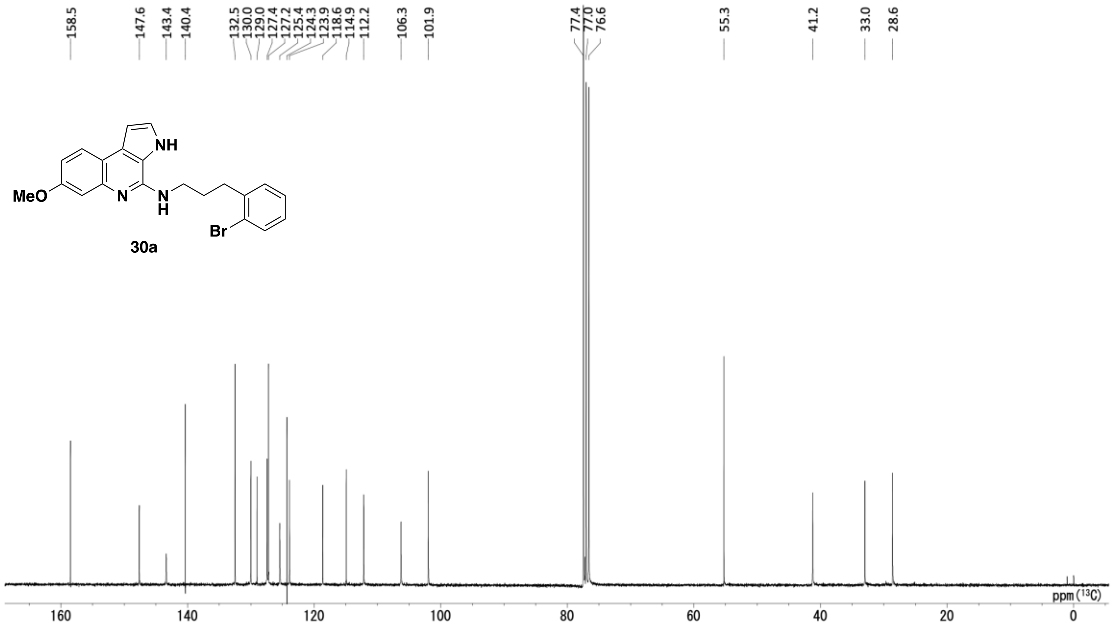

^{1}H NMR (300 MHz, $CDCl_3$) and ^{13}C NMR (75 MHz, $CDCl_3$) spectra of **30b**

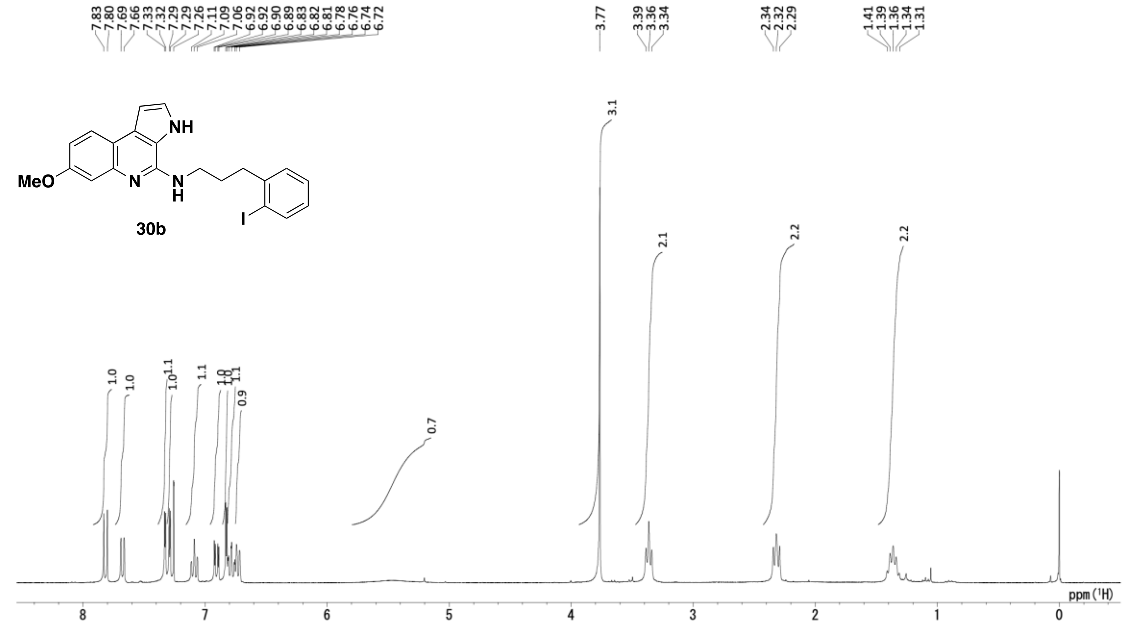

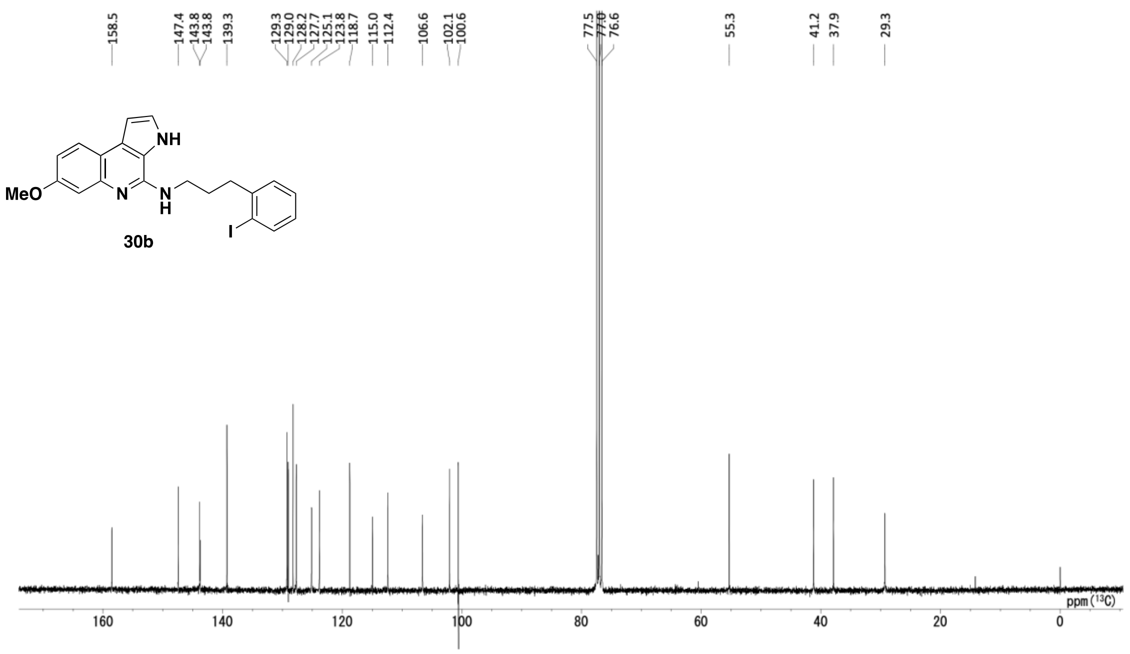

^{1}H NMR (300 MHz, $CDCl_3$) and ^{13}C NMR (75 MHz, $CDCl_3$) spectra of **31**

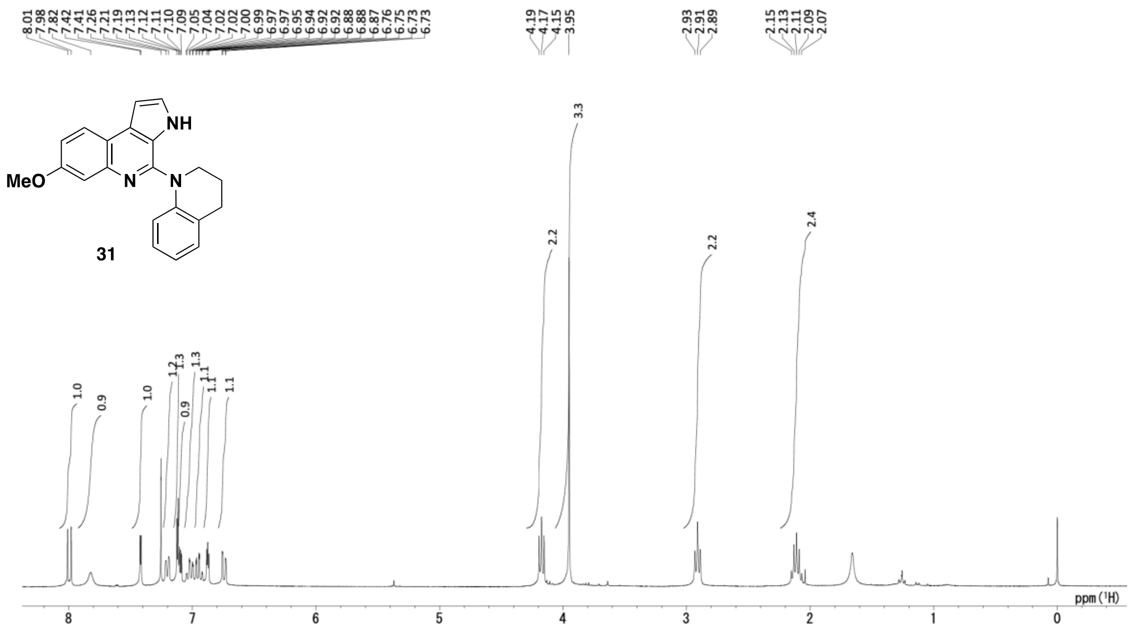

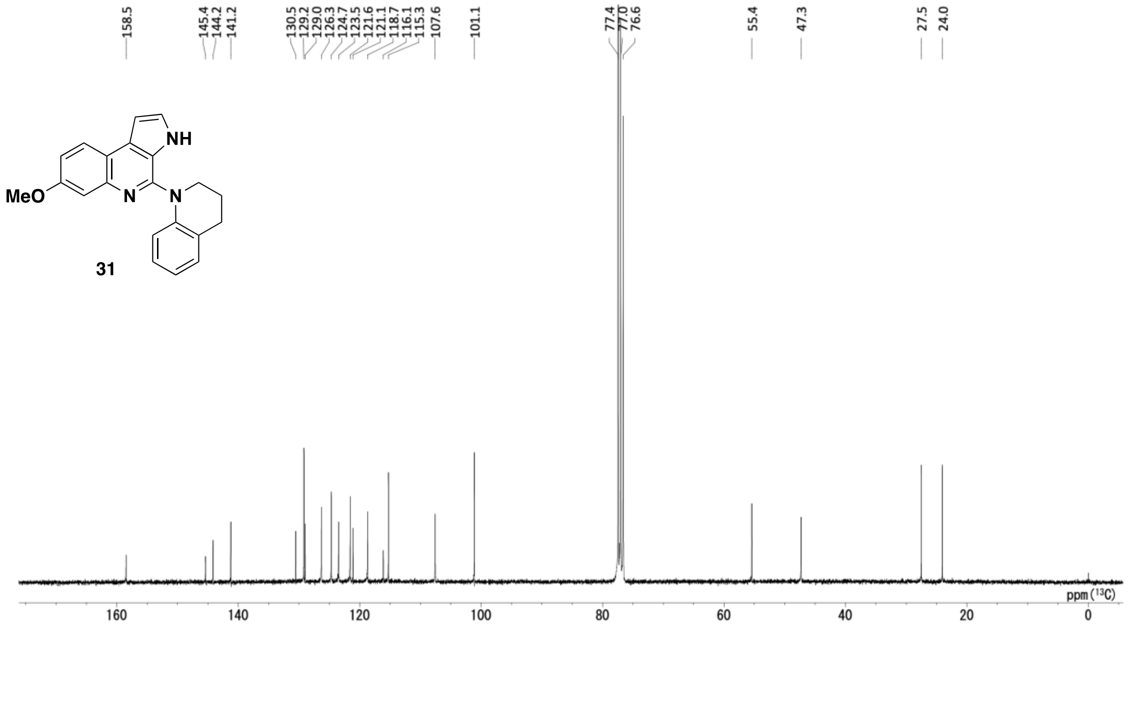

^{1}H NMR (300 MHz, DMSO-d_6) and ^{13}C NMR (75 MHz, DMSO-d_6) spectra of **1**

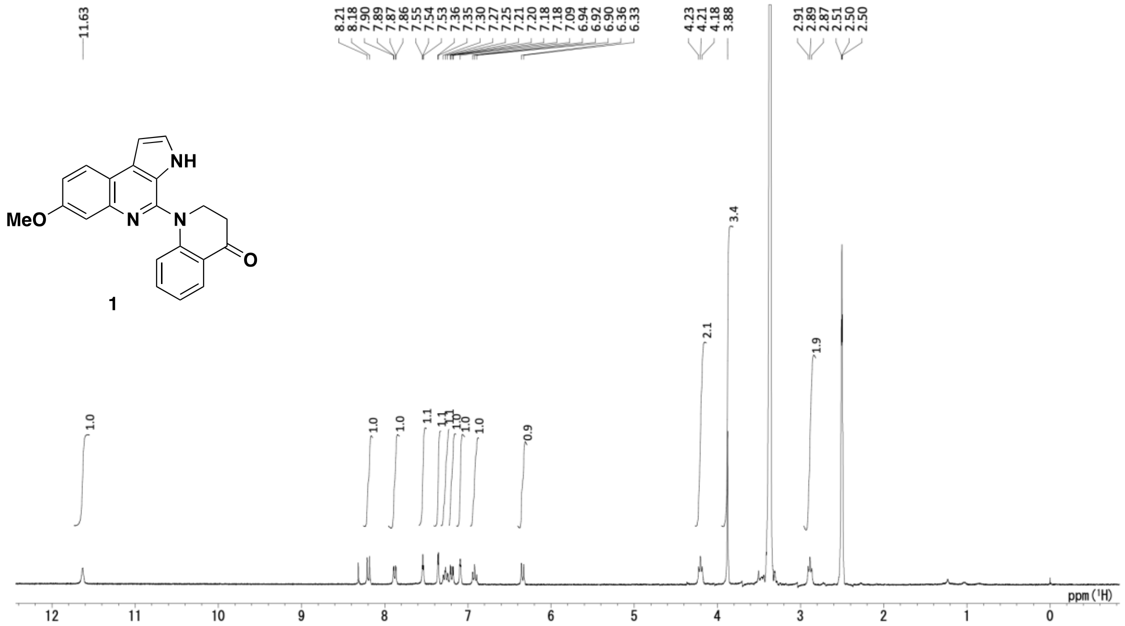

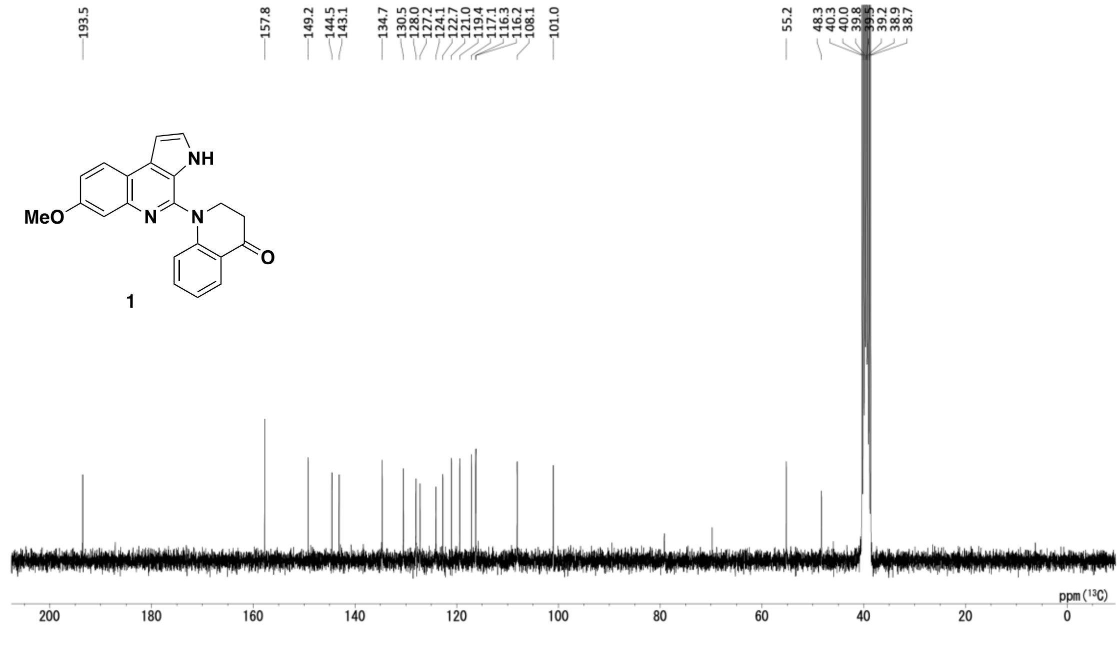

Supplement: File 1 — 1H NMR and 13C NMR spectra of all new compounds. [file Beilstein_J_Org_Chem-17-730-s001.pdf]
